# Multiple Linear Regression Predictive Modeling of Colloidal and Fluorescence Stability of Theranostic Perfluorocarbon Nanoemulsions

**DOI:** 10.3390/pharmaceutics15041103

**Published:** 2023-03-29

**Authors:** Michele Herneisey, Jelena M. Janjic

**Affiliations:** Graduate School of Pharmaceutical Sciences, School of Pharmacy, Duquesne University, Pittsburgh, PA 15282, USA; herneiseym@duq.edu

**Keywords:** ^19^F magnetic resonance imaging, near infrared fluorescence, design of experiments, multiple linear regression, perfluorocarbon nanoemulsion

## Abstract

Perfluorocarbon nanoemulsions (PFC-NEs) are widely used as theranostic nanoformulations with fluorescent dyes commonly incorporated for tracking PFC-NEs in tissues and in cells. Here, we demonstrate that PFC-NE fluorescence can be fully stabilized by controlling their composition and colloidal properties. A quality-by-design (QbD) approach was implemented to evaluate the impact of nanoemulsion composition on colloidal and fluorescence stability. A full factorial, 12-run design of experiments was used to study the impact of hydrocarbon concentration and perfluorocarbon type on nanoemulsion colloidal and fluorescence stability. PFC-NEs were produced with four unique PFCs: perfluorooctyl bromide (PFOB), perfluorodecalin (PFD), perfluoro(polyethylene glycol dimethyl ether) oxide (PFPE), and perfluoro-15-crown-5-ether (PCE). Multiple linear regression modeling (MLR) was used to predict nanoemulsion percent diameter change, polydispersity index (PDI), and percent fluorescence signal loss as a function of PFC type and hydrocarbon content. The optimized PFC-NE was loaded with curcumin, a known natural product with wide therapeutic potential. Through MLR-supported optimization, we identified a fluorescent PFC-NE with stable fluorescence that is unaffected by curcumin, which is known to interfere with fluorescent dyes. The presented work demonstrates the utility of MLR in the development and optimization of fluorescent and theranostic PFC nanoemulsions.

## 1. Introduction

**Figure 1:**
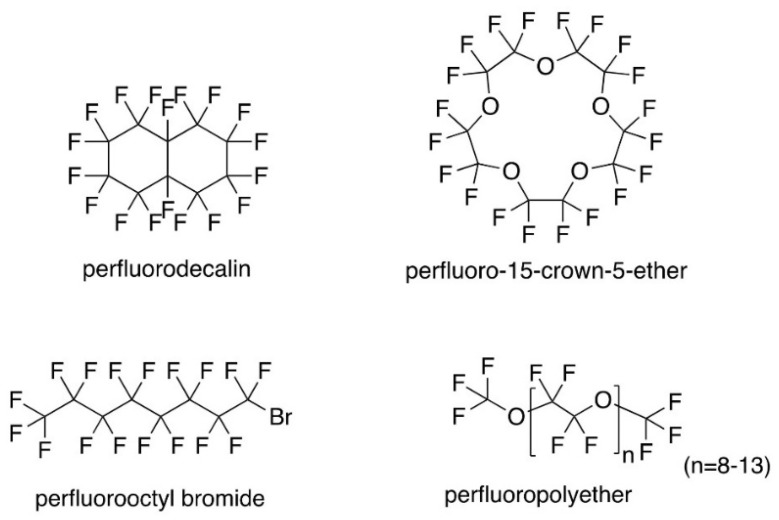
Chemical structures of the four perfluorocarbons selected for incorporation into nanoemulsions: perfluorodecalin (PFD), perfluoro-15-crown-5-ether (PCE), perfluorooctyl bromide (PFOB), and perfluoro(polyethylene glycol dimethyl ether) oxide (PFPE).

Perfluorocarbons are hydrocarbons which have had their hydrogen atoms replaced with fluorine atoms. Since C-F bonds are extremely strong and polar, this inhibits induced dipole formation, causing perfluorocarbons to demonstrate poor solubility in lipids [1]. Despite the extreme polarity of the individual C-F bonds, the symmetry of perfluorocarbon molecules (Figure 1) results in a net zero charge; therefore, the perfluorocarbon molecule itself is non-polar and is, thus, insoluble in water [1]. Therefore, perfluorocarbons possess the unique property of being both hydrophobic and lipophobic. When incorporated into an emulsion system, the perfluorocarbon will form a third, distinct fluorous phase. In nanoemulsions developed by our research group, the perfluorocarbon comprises the core of the nanoemulsion droplet. The perfluorocarbon core is surrounded by a hydrocarbon corona that can function as a depot for lipophilic drugs and fluorescent dyes. These resulting perfluorocarbon/hydrocarbon biphasic nanoemulsion droplets are stabilized in the third, continuous phase (water), using a mixture of non-ionic surfactants.

An attractive property of perfluorocarbons is that these compounds can be detected with ^19^F magnetic resonance imaging (MRI). Since organic fluorine is not naturally present in the human body, perfluorocarbons and perfluorocarbon nanoemulsions can be imaged with ^19^F MRI with no background interference [2,3,4]. Composite ^1^H (anatomical) and ^19^F MR (perfluorocarbon) images can be used to show perfluorocarbon location in vivo or ex vivo. Our group has previously developed celecoxib-loaded perfluorocarbon nanoemulsions for dual ^19^F MR and near infrared fluorescence (NIRF) imaging [5]. These nanoemulsions were used to image macrophage infiltration in response to chronic constriction injury (CCI) in rats. In this model, inflammation is induced by tying sutures around the sciatic nerve. Macrophage infiltration was successfully imaged in the inflamed sciatic nerve using NIRF in vivo, and these findings were confirmed ex vivo with ^19^F MRI. Further, since ^19^F MRI has unlimited penetration depth, the ^19^F MR analysis provided additional information that could not be detected with NIRF. Specifically, ^19^F signal was detected in the femur, suggesting that nanoemulsion was phagocytosed by monocytes present in the bone marrow [5]. This work highlighted the strengths of using nanoemulsions with dual imaging capabilities.

Another interesting property of perfluorocarbons is that these compounds are capable of dissolving large quantities of oxygen. Perfluorooctyl bromide (PFOB) and perfluorodecalin (PFD) are commonly used perfluorocarbons for oxygen delivery, as PFOB and PFD can dissolve 527 and 403 mL oxygen per liter, respectively [1]. These values are more than double that of blood, which can dissolve oxygen at 200 mL per liter [1]. For this reason, perfluorocarbons were initially developed as blood substitutes, with their first clinical use being reported in 1980 [6]. Lambert and Janjic recently reported the application of quality-by-design (QbD) approaches to identify parameters that have a significant impact on oxygen release from perfluorocarbon nanoemulsions in vitro [7]. The authors studied in vitro oxygen release in bi-phasic (perfluorocarbon-in-water) and tri-phasic (perfluorocarbon-in-hydrocarbon in water) nanoemulsions containing the perfluorocarbons PFOB and perfluoro-15-crown-5-ether (PCE). Increased oxygen release was associated with nanoemulsions with high perfluorocarbon content and/or low hydrocarbon content, while the perfluorocarbon type (PFOB or PCE) had no impact on oxygen release [7]. Given these findings, development of a triphasic perfluorocarbon nanoemulsion with maximum perfluorocarbon content and minimum hydrocarbon content should maximize the oxygen delivery potential of these formulations. Further, increased perfluorocarbon content would increase ^19^F MR sensitivity for the nanoemulsion. For these reasons, a primary goal of the work presented here was to identify stable perfluorocarbon nanoemulsions with high perfluorocarbon content and minimal hydrocarbon content. Lambert and Janjic reported tri-phasic nanoemulsions with a maximum perfluorocarbon content of 14.8% *w*/*v* [7]. In the presented work, we sought to double this content to 30% *w*/*v*.

As mentioned previously, earlier work by Vasudeva et al. demonstrated that macrophage infiltration to the inflamed sciatic nerve can be imaged with both NIRF and ^19^F MR using perfluorocarbon nanoemulsions tagged with a fluorescent reporter [5]. Incorporation of NIR fluorescent reporters into perfluorocarbon nanoemulsions is advantageous, as NIRF is a rapid and inexpensive method that can be used as a complementary method to ^19^F MRI. Previously, Balducci et al. demonstrated a high correlation between NIRF and ^19^F MRI when using fluorescently labeled tri-phasic (perfluorocarbon in hydrocarbon in water) perfluoro(polyethylene glycol dimethyl ether) oxide (PFPE) nanoemulsions to monitor monocyte infiltration to the tumor site in a murine subcutaneous breast carcinoma model [8]. However, decreased NIRF signal intensity and dissociation of the NIRF and ^19^F MR signals with time are major concerns surrounding the use of NIRF reporters. Bouvain et al. developed bi-phasic (perfluorocarbon in water) perfluorocarbon nanoemulsions coupled to rhodamine or carboxyfluorescein [9]. The authors found that fluorescence signal decreased over a 24 h period in vitro after uptake in multiple macrophage cell culture lines and in vivo after intravenous administration in a murine matrigel lipopolysaccharide (LPS) inflammation model, while ^19^F MR signal remained unaffected. Thus, when developing perfluorocarbon nanoemulsions with fluorescent dyes, it is critical to extensively evaluate the stability of the fluorescent reporter, both over the product’s shelf life and in response to relevant stresses, such as those experienced during in vitro cell culture labeling experiments.

In the presented work, QbD approaches were used to develop fluorescently labeled perfluorocarbon nanoemulsions with high perfluorocarbon content (30% *w*/*v*) and minimal hydrocarbon content (<10% *w*/*v*). Critical quality attributes (CQAs) were defined such that nanoemulsions that meet all CQA specifications will maintain acceptable colloidal and fluorescence stability upon storage and in response to stresses experienced during future cell culture work. Developed nanoemulsions were also subjected to accelerated stability tests designed to identify nanoemulsion formulations with unsuitable colloidal and/or fluorescence stability in a time-efficient manner. A total of 12 nanoemulsions were developed in a full factorial design of experiments (DoE) that evaluated the impact of perfluorocarbon type and hydrocarbon concentration on nanoemulsion colloidal and fluorescence stability. Three levels of hydrocarbon concentration (3, 6, and 9% *w*/*v*) and four perfluorocarbon types (PFOB, PFD, PFPE, and PCE) were evaluated. Through multiple linear regression (MLR) modeling, we identified the parameters that contribute to nanoemulsion colloidal and fluorescence stability, and we discovered that nanoemulsion destabilization is driven by different mechanisms dependent upon perfluorocarbon type. We also discovered that nanoemulsion colloidal and fluorescent stability are highly correlated. Using the insights gained from this first stage of work, a promising nanoemulsion candidate was selected to incorporate curcumin as a model drug. Nanoemulsion composition was optimized to maximize nanoemulsion colloidal, fluorescent, and drug-loading stability. The optimized formulation demonstrated superior stability under all accelerated stability testing conditions. To the best of our knowledge, the correlation between colloidal and fluorescent stability in perfluorocarbon nanoemulsions has not been reported. These findings are highly significant and should be used to inform development of future perfluorocarbon nanoemulsion formulations for macrophage targeting. 

## 2. Materials and Methods

### 2.1. Materials

Perfluorooctyl bromide (PFOB) was purchased from Exfluor, catalog number C8BR. Perfluorodecalin (PFD) was purchased from Sigma-Aldrich (Saint Louis, MO, USA), catalog number P9900-25G. Perfluoro(polyethylene glycol dimethyl ether) oxide (PFPE) was generously donated by Celsense, Inc., Pittsburgh, PA, USA. Perfluro-15-crown-5- ether (PCE) was purchased from Exfluor Research Company (Round Rock, TX, USA), product number F15CROWN5. Transcutol (2-(2-ethyoxyethyoxy)-ethanol (catalog E1022) and olive oil (catalog OL130) were purchased from Spectrum. Miglyol 812 was generously donated by Cremer Oleo (Hamburg, Germany), product number 6330. DMSO (dimethyl sulfoxide) was purchased from Acros Organics, catalog number 414885000. Fluorescent dyes were purchased from Invitrogen (Waltham, MA, USA). Catalog numbers for DiD (DiIC_18_(5), 1,1′-dioctadecyl-3,3,3′,3′-tetramethylindodicarbocyanine perchlorate), DiR (DiIC_18_(7), 1,1′-dioctadecyl-3,3,3′,3′-tetramethylindotricarbocyanine iodide), and DiI (DiIC_18_(3), 1,1′-dioctadecyl-3,3,3′,3′-tetramethylindocarbocyanine perchlorate) were D307, D12731, and D282, respectively. Cremophor EL was purchased from Sigma-Aldrich, catalog number C5135. Pluronic P105 was obtained from BASF Corporation (Florham Park, NJ, USA), material number 51207527.

### 2.2. Design of Experiments

JMP Pro13 software (JMP Group, San Francisco, CA, USA) was used to develop a full factorial design of experiments (DoE). The DoE consisted of two factors, a three-level continuous variable (olive oil concentration), and a four-level categorical variable (perfluorocarbon type). Olive oil concentration levels were defined as 3, 6, and 9% *w*/*v* of the total formulation. Perfluorocarbon type levels were defined as PFOB, PFD, PFPE, and PCE. This resulted in a DoE with 12 runs. Studied parameters included olive oil concentration, perfluorocarbon type, and the interaction between these two variables.

### 2.3. Nanoemulsion Production

Nanoemulsions were produced via sonication on a sonic dismembrator model 500 (Fisher Scientific, Waltham, MA, USA) followed by microfluidization on a microfluidizer M110S (Microfluidics, Westwood, MA, USA). Prior to nanoemulsion manufacture, curcumin solutions were prepared at a concentration of 100 mg/mL in Transcutol and fluorescent dye solutions were prepared at a concentration of 20 mM in DMSO. Nanoemulsion components were added in the following order: oil (miglyol or olive oil), curcumin solution (in transcutol), fluorescent dye solutions (in DMSO), perfluorocarbon, surfactant solution. Surfactant solution consisted of 3% *w*/*v* Cremophor EL and 2% *w*/*v* Pluronic P105 in water. The mixture was combined via vortexing for 30 s each time an excipient was added. Once all excipients were added, the resulting coarse emulsion was sonicated on ice for 30 s at 29% amplitude using a sonic dismembrator model 500 (Fisher Scientific, Waltham, MA, USA). Immediately after sonication, the emulsion was processed on a microfluidizer M110S (Microfluidics, Westwood, MA, USA) at 80 psi static nitrogen pressure for 20 pulses (four passes). Nanoemulsions were filtered through a 0.45 µm mixed cellulose esters membrane 24 h after production and stored at 4 °C until further testing or use in cell culture experiments.

### 2.4. Nanoemulsion Characterization

#### 2.4.1. Diameter and PDI Characterization

Nanoemulsion size distribution and polydispersity index (PDI) were evaluated using dynamic light scattering (DLS). Nanoemulsions were diluted in de-ionized water at 1:80 *v*/*v* prior to performing DLS measurements. All DLS measurements were performed in triplicate on a Zetasizer Nano ZS (Malvern Panalytical, Worcestershire, UK) at 25 °C and a light scattering angle of 173°.

#### 2.4.2. Fluorescence Characterization

Nanoemulsion fluorescence measurements were performed on a Tecan Infinite M1000 plate reader (Tecan, Männedorf, Switzerland). All fluorescence measurements were performed under the following settings: gain 100, flash frequency 400 Hz, 50 flashes, integration time 20 µs, lag time 0 µs, settle time 0 ms, and z-position 20,000 µm. Excitation and emission wavelengths and bandwidths were adjusted for each dye to achieve maximum fluorescence signal with minimal background signal. Settings for DiD measurements were: excitation wavelength 644 nm, emission wavelength 665 nm, excitation bandwidth 9 nm, and emission bandwidth 9 nm. Settings for DiR measurements were: excitation wavelength 750 nm, emission wavelength 780 nm, excitation bandwidth 13 nm, and emission bandwidth 13 nm. Settings for DiI measurements were: excitation wavelength 549 nm, emission wavelength 565 nm, excitation bandwidth 5 nm, and emission bandwidth 12 nm. All fluorescence measurements were performed using 96-well, black, flat-bottom, non-treated, polystyrene microplates (ThermoFisher, Nunc™ F96 MicroWell™, catalog number 237105). A volume of 100 µL was added to each well, and all measurements were performed in triplicate wells. Fluorescence measurements were taken for undiluted nanoemulsion and at several dilutions in de-ionized water (1:2, 1:4, 1:8, and 1:16 *v*/*v*) to confirm that observed nanoemulsion fluorescence trends (e.g., stability of fluorescence signal at elevated temperature) are consistent regardless of dilution factor.

### 2.5. Colloidal Stability Assessments

#### 2.5.1. Filtration Stability

Nanoemulsions were filtered through a Millex-GS syringe filter with a pore size of 0.22 μm approximately 2 h after manufacture. Filtered nanoemulsion was diluted 1:80 *v*/*v* in de-ionized water, and diameter and PDI were measured using DLS at 25 °C with a light scattering angle of 173°. Same-day measurements of unfiltered nanoemulsions were used as a control.

#### 2.5.2. Cell Culture Conditions

Nanoemulsions were diluted 1:80 *v*/*v* in Dulbecco’s modified Eagle’s medium (DMEM) containing 10% fetal bovine serum (FBS). Dilutions in cell culture medium were incubated for 3 h at 37 °C. After incubation, nanoemulsions were centrifuged at 1100 rpm for 5 min (Eppendorf 5804R, Framingham, MA, USA). After centrifugation, sample diameter and PDI were measured using DLS at 25 °C with a light scattering angle of 173°. Same-day measurements of nanoemulsion stored at 4 °C were used as a control. 

#### 2.5.3. Accelerated Stability at Elevated Temperature

500 µL of undiluted nanoemulsion were added to a 1.5 mL Eppendorf tube. These accelerated stability samples were placed into beakers containing a small amount of water, and the beakers were covered with aluminum foil to prevent nanoemulsion water loss. Samples were incubated for 7 days at 80 °C (DoE formulations) or 50 °C (curcumin formulations). Water was added to the beakers as necessary throughout the study. The Eppendorf tubes containing the nanoemulsion were weighed at the beginning and end of the study to confirm that water loss had not occurred during the study. Upon incubation completion, samples were allowed to equilibrate to room temperature for 1 h. Nanoemulsions were diluted 1:80 *v*/*v* in de-ionized water, and diameter and PDI were measured with DLS at 25 °C and a light scattering angle of 173°.

#### 2.5.4. Shelf-Life Stability

Nanoemulsion diameter was measured on days 1, 95, and 215 after production. Nanoemulsions were diluted 1:80 *v*/*v* in de-ionized water, and diameter and PDI were measured with DLS at 25 °C and a light scattering angle of 173°. All measurements were performed in triplicate. Shelf-life stability was reported in terms of percent diameter change over the 95- or 215-day period. 

### 2.6. Fluorescence Stability Assessments

#### 2.6.1. Cell Culture Conditions

Nanoemulsion was diluted 1:5 *v*/*v* in cell culture media (DMEM containing 10% FBS), then incubated at 37 °C for a period of 2 or 12 h. After the incubation period, fluorescence was measured without further sample dilution using the Tecan Infinite M1000 under the settings described above (Section 2.4.2). As a control, nanoemulsions were diluted 1:5 *v*/*v* in cell culture media, and fluorescence signal was measured immediately after dilution (no incubation period). Stability under cell culture conditions was reported in terms of percent fluorescence signal loss. 

#### 2.6.2. Accelerated Stability at Elevated Temperature

To evaluate nanoemulsion fluorescence stability, undiluted nanoemulsions were stored at 25 °C or 37 °C for 24 or 72 h to mimic conditions that formulations may experience upon shipping (25 °C) or in vitro cell culture testing (37 °C). After this time period, nanoemulsion samples were diluted in de-ionized water at 1:2, 1:4, 1:8, and 1:16 *v*/*v*. Fluorescence measurements of these dilutions, as well as undiluted nanoemulsion, were obtained using the Tecan Infinite M1000 under the settings described above (Section 2.4.2). Same-day measurements of nanoemulsions stored at 4 °C were used as a control. Stability at elevated temperature was reported in terms of percent fluorescence signal loss. 

#### 2.6.3. Shelf-Life Stability

Nanoemulsion fluorescence was measured on days 1, 95, and 215 after production. To perform fluorescence measurements, a volume of 100 µL was added to each well of the 96-well plate. All measurements were performed in triplicate. Fluorescence measurements were taken for undiluted nanoemulsion and at several dilutions in de-ionized water (1:2, 1:4, 1:8, and 1:16 *v*/*v*) to confirm that observed nanoemulsion fluorescence trends are consistent regardless of dilution factor. Shelf-life stability was reported in terms of percent fluorescence signal loss over the 95- or 215-day period

### 2.7. Determination of Nanoemulsion Perfluorocarbon Loading with ^19^F NMR

^19^F NMR was performed on 400 MHz, 52 mm NMR (Bruker, MA, USA). NMR samples were prepared by combining 200 μL nanoemulsion, 200 μL of an aqueous trifluoroacetic acid (TFA) solution (0.4% *v*/*v* TFA), and 50 µL deuterium oxide (D_2_O). Samples were scanned 16 times. TFA contains three chemically equivalent fluorine atoms that result in a peak at −76.0 ppm. Using the molecular weight (114.02 g/mol) and the density (1.489 g/mL) of TFA, it was calculated that an NMR sample containing 200 µL of a 0.4% *v*/*v* TFA solution contains 6.29 × 10^18^ molecules of TFA, or 1.89 × 10^19^ atoms of fluorine. Similarly, perfluorocarbon densities, molecular weights, and known numbers of chemically equivalent fluorine atoms at a specific NMR peak can be used to determine the number of perfluorocarbon molecules that should be present in the NMR sample if the nanoemulsion is 100% perfluorocarbon loading. Table 1 summarizes these values.

After collection of NMR spectra, TFA and perfluorocarbon peak integration areas were calculated using TopSpin 3.6.4 software (Bruker, Billerica, MA, USA). These integration areas were used to calculate the number of perfluorocarbon molecules in the NMR sample. Subsequently, the calculated amount of perfluorocarbon molecules in the NMR sample and the theoretical number of perfluorocarbon molecules that should be in the sample if the sample were 100% perfluorocarbon loading were used to calculate nanoemulsion percent perfluorocarbon loading and nanoemulsion percent sedimentation values. 

### 2.8. Multiple Linear Regression Modeling

All multiple linear regression (MLR) was performed using JMP Pro13 statistical software. MLR models were generated using a standard least squares approach. A backward stepwise regression approach was used to eliminate variables that did not have a significant impact on the output of interest. Studied terms included the main effects of olive oil concentration and perfluorocarbon type, as well as the interaction between olive oil concentration and perfluorocarbon type. In the shelf life MLR models, the additional term of percent fluorescence signal loss over the storage period was included in the shelf-life models for percent diameter change, and the additional term of percent diameter change over the storage period was included in the shelf life models for percent fluorescence signal loss. Terms with a *p*-value < 0.05 were included in the final model.

### 2.9. Assessment of Nanoemulsion Curcumin Loading and Stability

Nanoemulsion curcumin concentration was measured using a previously reported HPLC method [12]. Standard curves of curcumin in 1:1 acetonitrile: water (concentration range 0.1–20 µg/mL) were prepared in triplicate (Supplemental Figure S2), and these standard curves were used to quantify curcumin loading samples. To prepare samples, nanoemulsions were diluted with a 1:1 solution of acetonitrile and water to a theoretical concentration of 40 µg/mL and vortexed to break the nanoemulsion. The resultant dilutions were centrifuged to separate perfluoro-15-crown-5-ether (PCE), which is insoluble in the acetonitrile: water solution. PCE has a density of 1.78 g/mL, and, therefore, separates to the bottom upon centrifugation. The resulting supernatants were evaluated for curcumin concentration using HPLC with a flow rate of 1.2 mL/minute, column oven temperature 33 °C, absorption wavelength 425 nm, and injection volume 50 µL. An isocratic mobile phase of 60% acetonitrile and 40% water (acidified with 2% *v*/*v* acetic acid) was used. The column was a 150 × 4.6 mm gold C18 column with a 5 µm pore size (Hypersil Gold C18, 25,005–154,630, Thermo Fisher, Vilnius, Lithuania). Curcumin elution occurred at approximately 2.6 min. To evaluate curcumin loading and curcumin stability, undiluted nanoemulsion was incubated at 37 °C for 72 h. The methods described above were used to measure nanoemulsion curcumin concentration after the 72 h incubation. As a control, same-day measurements were obtained for curcumin nanoemulsions stored at 4 °C throughout the study. 

## 3. Results

### 3.1. Study Design and Identification of Critical Quality Attribute and Quality Control Testing Conditions

In previous studies, our group was able to successfully model the colloidal stability of microemulsions in terms of percent diameter change after 30 days storage [13], and nanoemulsions in terms of percent diameter change over 90 days storage [14]. In these earlier studies, formulations were stored at ambient temperature. Thus, 30 days or longer of storage was required before accurate models could be developed to predict formulation stability. Ideally, accelerated stability testing could be employed to identify the parameters that have a significant impact on nanoemulsion colloidal stability, as this would allow for identification of suitable candidates for further testing and optimization in a time-efficient manner. Thus, in the presented work, nanoemulsions were subjected to incubation at 80 °C for 7 days as a form of accelerated stability testing (Table 2). In a clinical setting, nanoemulsions would not be subjected to this harsh condition. For this reason, this test was defined as a quality control (QC) test.

For accelerated stability model predictions to be meaningful, they must accurately reflect the behavior of the formulations under non-accelerated (storage) conditions. To test this, nanoemulsions in the presented study were also evaluated for percent diameter change and PDI after 95 days (~3 months) and 215 days (~7 months) storage at 4 °C (Table 2). Nanoemulsion colloidal stability upon storage is essential to final product efficacy, so percent diameter change and PDI after 95 days storage at 4 °C were defined as CQAs. Nanoemulsion stability beyond 6 months is preferable, but not required, so nanoemulsion percent diameter change and PDI after 215 days storage at 4 °C were defined as QC parameters. This extended shelf-life evaluation was included as an additional time point that could be used to evaluate the predictive relevancy of the accelerated stability models. 

To be tested in vitro and in vivo, it is necessary that nanoemulsion diameter and PDI are not impacted by filtration through a 0.22 µm filter, as this is the method of sterilization for these formulations. Further, it is essential that nanoemulsions maintain colloidal stability upon exposure to conditions encountered during cell culture. Therefore, nanoemulsion percent diameter change and PDI upon filtration and exposure to cell culture conditions were defined as CQAs (Table 2). Specifically, exposure to cell culture conditions consisted of nanoemulsion dilution at 1:80 *v*/*v* in cell culture media and incubation of the resulting dilution at 37 °C for 3 h, followed by centrifugation at 1100 rpm for 5 min. This sequence of testing conditions was selected because it simulates conditions nanoemulsions could experience upon ex vivo cell labeling, a potential application of these products [10]. All diameter and PDI CQA and QC testing descriptions and specifications are summarized in Table 2.

CQAs, CQA specifications, and QC testing conditions were also identified for nanoemulsion fluorescence stability (Table 3). Fluorescence CQAs were defined as the percent fluorescence signal loss upon (1) incubation of undiluted nanoemulsion at 25 °C for 24 h, (2) incubation of nanoemulsion diluted in cell culture media at 37 °C for 2 h, or (3) storage at 4 °C for 95 days. Incubation at 25 °C for 24 h was defined to account for times that the nanoemulsion may be left at ambient temperature during transportation or on a benchtop prior to use. Incubation in cell culture media at 37 °C for 2 h was defined to mimic conditions nanoemulsions will experience upon use in vitro in cell culture assays. The nanoemulsions are designed to passively target macrophages, which phagocytose nanoemulsion droplets within 2 h of exposure in cell culture. As forms of accelerated stability testing, percent fluorescence signal loss was also assessed after incubation of undiluted nanoemulsion at 25 °C for 72 h, and after incubation of nanoemulsion diluted in cell culture media at 37 °C for 12 h. These extended incubation times were defined as QC tests (Table 3). Additionally, nanoemulsion percent fluorescence signal loss after 215 days storage at 4 °C was included as a QC test, so that fluorescence signal loss could be assessed in parallel with nanoemulsion percent diameter change over the shelf life of the product.

As mentioned in the introduction, the primary goals of this work were to identify stable nanoemulsions with high perfluorocarbon loading (30% *w*/*v*) and minimal hydrocarbon concentration, and to understand the parameters that have an impact on the stability of the fluorescent reporter in these complex nanoemulsion formulations. To achieve these goals, a full factorial design of experiments (DoE) was developed to study the impact of nanoemulsion perfluorocarbon type and hydrocarbon concentration on nanoemulsion colloidal and fluorescent stability (Table 4). Three levels were defined for hydrocarbon concentration (3, 6, and 9% *w*/*v*), and four levels were defined for perfluorocarbon type (PFOB, PFD, PFPE, and PCE), resulting in a 12-run full factorial design. Nanoemulsion formulations are summarized in Table 4, and perfluorocarbon structures are shown in Figure 1. Nanoemulsions were developed using minimal processing (30 s sonication and four passes on the microfluidizer), as previous work [14] demonstrated that nanoemulsion processing, particularly sonication time, can decrease nanoemulsion drug encapsulation efficiency. Thus, in this work, processing conditions were limited to maximize incorporation of the fluorescent reporter (DiD). 

### 3.2. Assessment of Nanoemulsion Colloidal and Fluorescence Stability with Multiple Linear Regression (MLR) Modeling

All developed nanoemulsions were subjected to the colloidal and fluorescence stability CQA and QC tests summarized in Table 2 and Table 3. The results of the CQA testing are summarized in Supplemental Table S1 and the results of the QC testing are summarized in Supplemental Table S2. The majority of nanoemulsion formulations (eight of twelve) met all colloidal CQA specifications. These include baseline diameter and PDI, as well as nanoemulsion percent diameter change and PDI after filtration, after exposure to cell culture conditions, and after 95 days storage at 4 °C (Supplemental Table S1). Three of the four nanoemulsions that failed to meet one or more colloidal CQA specifications contained 3% *w*/*v* olive oil, suggesting that olive oil concentrations higher than 3% *w*/*v* are required to achieve colloidally stable nanoemulsions with 30% *w*/*v* perfluorocarbon, regardless of perfluorocarbon type. An unexpected finding was that PFOB and PFD nanoemulsions at all olive oil concentrations decrease in diameter over 95 days storage at 4 °C, as shown by negative percent diameter change values. This decrease in diameter (negative percent diameter change value) is also observed for all PFOB and PFD nanoemulsions after incubation at 80 °C for 7 days, and after 215 days storage at 4 °C (Supplemental Table S2). 

**Figure 2:**
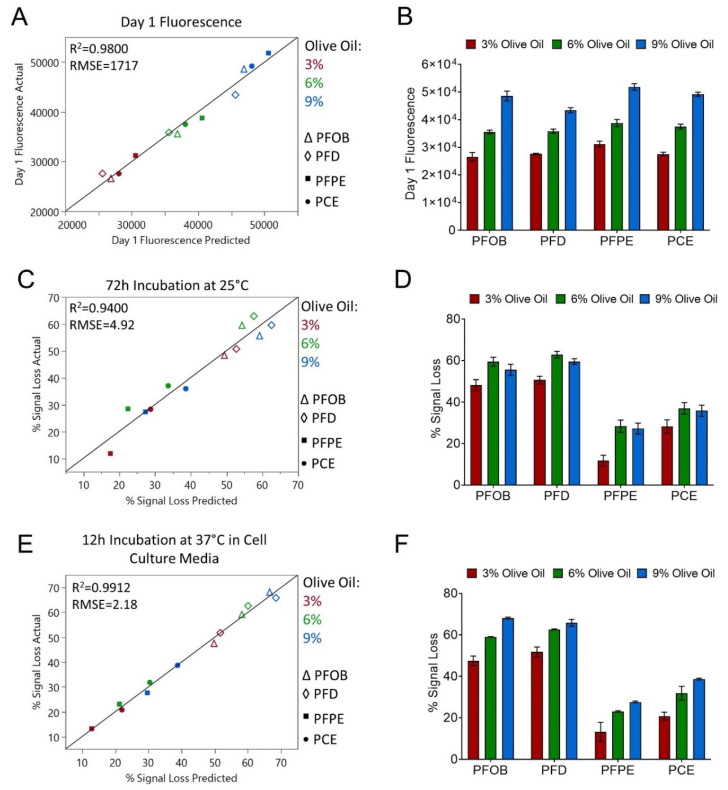
MLR models were developed to predict (**A**,**B**) baseline fluorescence (24 h after production) and percent fluorescence signal loss after (**C**,**D**) 72 h incubation at 25 °C and (**E**,**F**) 12 h incubation in cell culture media at 37 °C.

Another unexpected finding was that that vast majority of nanoemulsions failed to meet one or more fluorescence CQA specification (Supplemental Table S1). Previously developed tri-phasic, perfluorocarbon nanoemulsions containing NIRF dyes have been evaluated in a murine model of complete Freund’s adjuvant (CFA) induced inflammation [15], a murine subcutaneous breast carcinoma model [8], and a rat CCI model [5]. In these studies, significant loss of fluorescence signal was not observed, and fluorescence signal could be detected in vivo 40 days after a single i.v. nanoemulsion administration [15], suggesting superior stability of the NIRF dye in vivo. To understand the parameters driving fluorescence signal loss in the presented formulations, MLR models were developed to predict nanoemulsion baseline (day 1) fluorescence signal, nanoemulsion fluorescence signal loss after 72 h incubation at 25 °C, and nanoemulsion percent fluorescence signal loss after 12 h incubation in cell culture media at 37 °C (Figure 2, Table 5).

Olive oil concentration is the most significant parameter driving baseline fluorescence signal, with a model *p*-value < 0.0001 (Table 5). The actual vs. predicted plot highlights this, as three clusters of formulations grouped by oil type are observed (Figure 2A), and the bar plot shows increasing fluorescence signal with increasing olive oil concentration across all perfluorocarbon types (Figure 2B). Perfluorocarbon type was also found to have a significant impact on baseline fluorescence (*p*-value 0.04487). The scaled coefficients for the PFD and PFOB terms are negative, indicating that nanoemulsions containing these perfluorocarbons have a decreased baseline fluorescence signal compared to nanoemulsions containing PFPE and PCE that is independent of olive oil concentration (Table 5). With the largest positive scaled coefficient, nanoemulsions containing PFPE were determined to have the highest baseline fluorescence signal (Table 5). 

MLR models developed to predict nanoemulsion percent fluorescence signal loss after 72 h incubation at 25 °C or after 12 h incubation in cell culture media at 37 °C are comparable (Figure 2, Table 5). Similar conclusions can be drawn from both models, suggesting that nanoemulsions respond similarly to the two different stressors. Perfluorocarbon type was found to significantly contribute to both models, with a model *p*-value of <0.00001 for the 37 °C model and a *p*-value of 0.00014 for the 25 °C model. In both models, scaled coefficients indicate that fluorescence signal loss is greater in nanoemulsions containing PFOB and PFD (Table 5). The actual vs. predicted plots highlight this, as distinct separations can be observed dividing PFOB and PFD nanoemulsions from PFPE and PCE nanoemulsions (Figure 2C,E). Bar plots also show that fluorescence signal loss is greater in PFOB and PFD nanoemulsions (Figure 2D,F). 

Olive oil concentration was also found to significantly contribute to fluorescence signal loss, with a *p*-value < 0.0001 in the 37 °C model and a *p*-value of 0.0254 in the 25 °C model (Table 5). Scaled coefficients for the olive oil concentration terms are positive in both models, indicating that increasing olive oil concentration results in a greater loss of fluorescence signal. This trend is particularly apparent in the 37 °C model (Figure 2F). Together, the fluorescence models demonstrate that increasing olive oil concentration will increase nanoemulsion baseline fluorescence signal, but at a cost, as increasing olive oil concentration results in greater fluorescence signal loss in response to stresses (i.e., elevated temperature and dilution in cell culture media). PFPE and PCE nanoemulsions appear more suitable for the incorporation of fluorescent dyes, as nanoemulsions containing these perfluorocarbons had higher baseline fluorescence signal and lower fluorescence signal loss in response to stress when compared to nanoemulsions containing PFOB and PFD.

**Figure 3:**
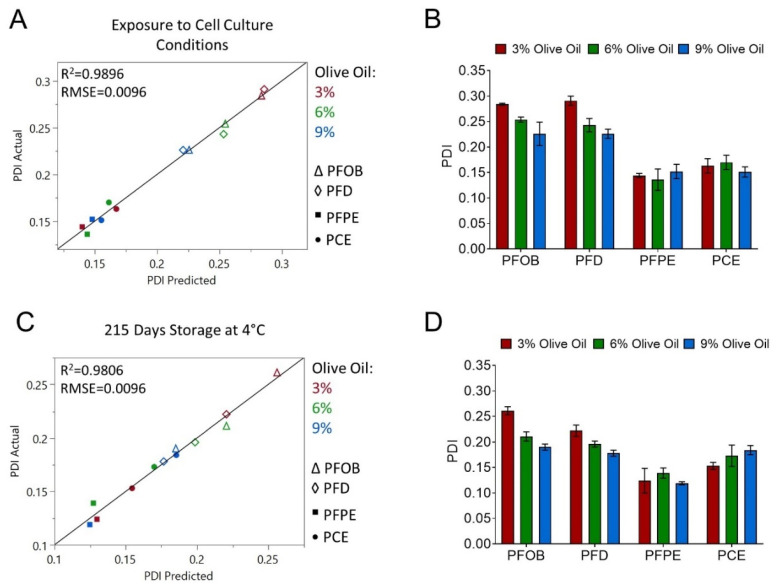
MLR models were developed to predict (**A**,**B**) nanoemulsion PDI after exposure to cell culture conditions and (**C**,**D**) after 215 days storage at 4 °C.

After investigating the parameters that influence baseline fluorescence signal and fluorescence stability, MLR models were developed to understand the impact of olive oil concentration and perfluorocarbon type on nanoemulsion PDI after exposure to stress (exposure to cell culture conditions) and after extended storage (215 days at 4 °C). These two models were developed in parallel to assess whether results of the stress stability test could be used to accurately predict nanoemulsion behavior upon long-term storage. Model results are shown in Figure 3 and Table 6. 

The cell culture conditions test was designed to mimic the conditions that nanoemulsions could be exposed to upon ex vivo labeling conditions. This test is described in Section 2.5.2. An accurate MLR model was developed to predict nanoemulsion PDI after exposure to cell culture conditions (Figure 3A,B, Table 6). Perfluorocarbon type (*p*-value 0.00025) and olive oil concentration (*p*-value 0.00951) both significantly contribute to nanoemulsion PDI after exposure to cell culture conditions, and a significant interaction between perfluorocarbon type and olive oil concentration was detected (*p*-value 0.04763). The actual vs. predicted plot (Figure 3A) and bar plot (Figure 3B) show that nanoemulsions containing PFOB and PFD have higher PDI values than nanoemulsions with PFPE and PCE. Accordingly, the scaled coefficients for perfluorocarbon type are positive for PFOB and PFD and negative for PFPE and PCE (Table 6). Olive oil concentration has a negative scaled coefficient estimate, indicating that increasing olive oil concentration reduces nanoemulsion PDI in response to cell culture conditions. This effect is most apparent for PFOB and PFD nanoemulsions (Figure 3A,B). 

The MLR model for PDI after 215 days storage at 4 °C was comparable to the model for PDI after exposure to cell culture conditions in several ways. Perfluorocarbon type (*p*-value 0.00112), olive oil concentration (*p*-value 0.03055) and an interaction between these two parameters (*p*-value 0.02171) all contributed significantly to PDI after 215 days storage (Table 6). Scaled coefficient values indicate that increasing olive oil concentration reduces PDI, and that nanoemulsions with PFOB and PFD have larger PDI values compared to nanoemulsions containing PFPE and PCE. These trends can be seen in the model actual vs. predicted plot (Figure 3C) and in the bar plot of PDI values (Figure 3D). Therefore, the MLR model for PDI after exposure to cell culture conditions was able to estimate nanoemulsion PDI trends upon long-term storage. 

**Figure 4:**
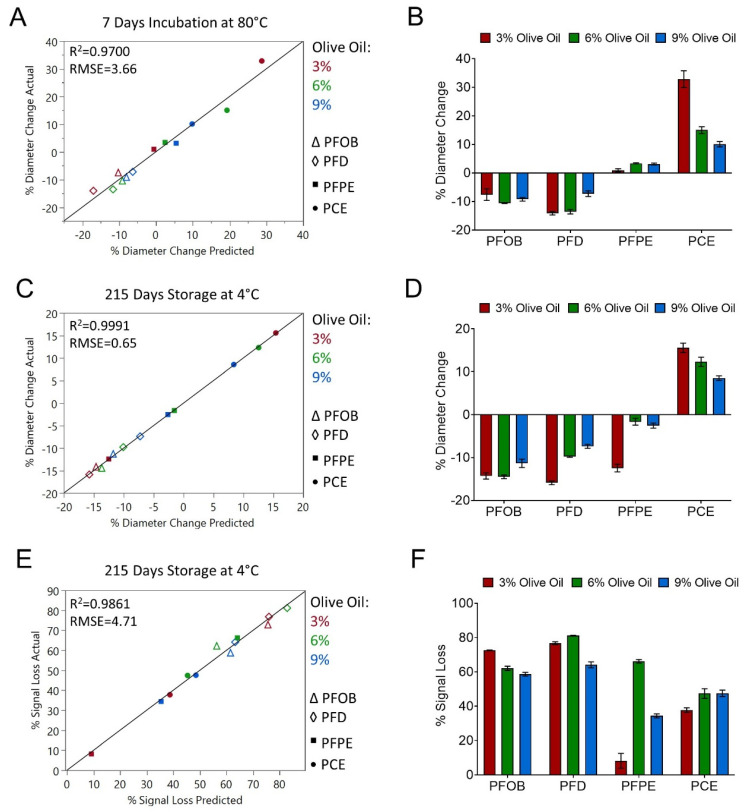
MLR models were developed to predict (**A**,**B**) nanoemulsion percent diameter change after 7 days incubation at 80 °C, (**C**,**D**) nanoemulsion percent diameter change after 215 days storage at 4 °C, and (**E**,**F**) nanoemulsion percent fluorescence signal loss after 215 days storage at 4 °C.

High PDI values indicate that a nanoemulsion has a broader droplet diameter range, which can decrease nanoemulsion colloidal stability. Thus, together these data suggest that nanoemulsions containing PFOB and PFD may be less stable compared to nanoemulsions containing PFPE and PCE. This possibility was further investigated by developing MLR models to predict nanoemulsion percent diameter change after 7 days incubation at 80 °C (Figure 4A,B, Table 7) and after 215 days storage at 4 °C (Figure 4C,D, Table 7). Similar to PDI, an accelerated stability model and a shelf-life stability model were developed in parallel to assess whether results of the accelerated stability test could be used to accurately predict nanoemulsion behavior upon long-term storage. Nanoemulsion percent diameter change after 7 days storage at 80 °C was found to be dependent on perfluorocarbon type (*p*-value 0.0005) and the interaction between olive oil concentration and perfluorocarbon type (*p*-value 0.0367). Interestingly, incubation at 80 °C resulted in a reduction in average nanoemulsion diameter for nanoemulsions containing PFOB and PFD (Figure 4A,B, Table 7). Average diameter of PFPE nanoemulsions remained relatively unaffected over the incubation period, while average diameter of PCE nanoemulsions increased. This increase was significantly dependent upon olive oil concentration, as the interaction term between olive oil concentration and PCE had a *p*-value of 0.0083. This suggests that increasing olive oil concentration may improve the colloidal stability of PCE nanoemulsions.

An MLR model was next developed to predict nanoemulsion percent diameter change over 215 days storage at 4 °C (Figure 4C,D, Table 7). An accurate model could not be developed solely using the terms of olive oil concentration, perfluorocarbon type, and their interaction. However, upon inspection of the models developed thus far, it appeared that a possible correlation may exist between nanoemulsion colloidal stability and nanoemulsion fluorescence stability. To investigate this possibility, the percent fluorescence signal loss over 215 days storage at 4 °C was included as an additional predictor variable in the MLR model for percent diameter change over 215 days storage at 4 °C. Upon addition of this term, an accurate MLR model was developed (Figure 4C,D, Table 7). Percent fluorescence signal loss over 215 days storage (*p*-value 0.0042) significantly contributed to nanoemulsion percent diameter change over 215 days storage. Olive oil concentration (*p*-value 0.0057), perfluorocarbon type (*p*-value 0.0001), and the interaction between these two variables (*p*-value 0.0025) also significantly contributed to nanoemulsion percent diameter change over 215 days storage. Importantly, the impacts of storage at 4 °C and incubation at 80 °C on nanoemulsion percent diameter change were comparable. Prolonged storage at 4 °C resulted in a decreased diameter in nanoemulsions containing PFOB and PFD and an increased diameter in PCE nanoemulsions. Nanoemulsions containing PFPE were the most resistant to changes in diameter upon storage at 4 °C (Figure 4C,D). The interaction between olive oil concentration and PCE indicated that increasing olive oil concentration improves nanoemulsion colloidal stability by reducing nanoemulsion percent diameter change upon storage at 4 °C. In summary, exposing nanoemulsions to elevated temperature as a form of accelerated stability testing was determined to accurately predict nanoemulsion colloidal stability upon long-term storage at 4 °C. A correlation between nanoemulsion colloidal stability and nanoemulsion fluorescence stability was also established. 

To further investigate the relationship between colloidal and fluorescence stability, an MLR model was developed to predict nanoemulsion percent fluorescence signal loss after 215 days storage at 4 °C (Figure 4E,F, Table 7). As was observed when modeling percent diameter change after 215 days storage, an accurate model could not be developed solely using the terms of olive oil concentration, perfluorocarbon type, and their interaction. When percent nanoemulsion diameter change after 215 days storage was included as an additional predictive term, an accurate model was developed. As was observed with the prolonged storage model of percent diameter change, fluorescence loss was found to be dependent upon olive oil concentration (*p*-value 0.0155), perfluorocarbon type (*p*-value 0.0044), and their interaction (*p*-value 0.0134). Nanoemulsions containing PFOB and PFD experienced greater fluorescence signal loss upon prolonged storage at 4 °C when compared to nanoemulsions containing PFPE and PCE. These observations are comparable with those from the accelerated models of fluorescence stability (Figure 2C,F), suggesting that the accelerated stability models are a good predictor of how nanoemulsions will behave over their shelf life.

### 3.3. Evaluation of Nanoemulsion Sedimentation with ^19^F Nuclear Magnetic Resonance (NMR)

In summary thus far, we identified accelerated stability tests that could be used to understand the impact of olive oil concentration and perfluorocarbon type on nanoemulsion colloidal and fluorescence stability in a time-efficient manner. Relationships detected under these accelerated conditions corresponded to nanoemulsion behavior upon prolonged (215 days) storage at 4 °C. Thus, results of the accelerated stability testing can be used to predict nanoemulsion shelf-life behavior. Further, we determined that there is a correlation between nanoemulsion colloidal stability and nanoemulsion fluorescence stability. We determined that PFOB and PFD nanoemulsions consistently underperform when compared to the PFPE and PCE nanoemulsions. As the next step in this work, we sought to develop an explanation for the clear distinctions observed between these two “groups” of nanoemulsions. While working with the nanoemulsions presented in this work, it was observed that if the nanoemulsions were left undisturbed, a thick white opaque layer would form on the bottom of the container. This observation was most apparent in nanoemulsions containing PFOB and PFD, though it was observed in all developed nanoemulsions. Upon gentle agitation, the sedimentation layer would become redispersed, and the nanoemulsion would regain a homogenous appearance. These observations were not surprising when considering the densities of the perfluorocarbons studied in this work, which range from 1.75 to 1.93 g/mL (Table 1). Since the developed nanoemulsions contain high levels of perfluorocarbon (30% *w*/*v*), the density between the dispersed phase (nanoemulsion droplet) and the continuous phase (water) is significant, which can accelerate sedimentation. PFOB and PFD have comparable densities, at 1.92 and 1.93 g/mL, respectively, while PFPE and PCE have comparable densities, at 1.75 and 1.78 g/mL, respectively (Table 1). Given this, we hypothesized that nanoemulsions containing PFOB and PFD experience sedimentation at a faster rate when compared to nanoemulsions containing PFPE or PCE. Nanoemulsion droplet sedimentation and the resulting colloidal instability leads to loss of fluorescence signal. The proposed mechanism could explain the observed correlations between nanoemulsion colloidal properties and percent fluorescent signal loss. Further, this mechanism could provide an explanation as to why the majority of the nanoemulsions presented in this work failed to meet one or more CQA specification for fluorescence (Supplemental Table S1), while nanoemulsions previously studied by our research group demonstrated superior fluorescence stability in vitro and in vivo [5,8,15]. These previously studied nanoemulsions had higher hydrocarbon content and lower perfluorocarbon content compared to the nanoemulsions studied in this work; thus, the dispersed phase of the previously studied nanoemulsions [5,8,15] had a lower density, making these formulations less susceptible to sedimentation. 

To test this hypothesis, nanoemulsions were left undisturbed at 4 °C for 20 days. After this time, a small volume (300 µL) was removed from the top of the nanoemulsion container, above any observed sediment. Nanoemulsions were then gently agitated to resuspend any sedimentation, and an additional 300 µL were removed. Nanoemulsion samples obtained before and after the resuspension were evaluated for diameter and PDI using DLS, and for fluorine content using ^19^F NMR. Diameter and PDI values measured before and after resuspension were used to calculate the percent diameter change and the change in PDI after resuspension (Table 8). Fluorine content was quantified in the samples obtained before and after resuspension, and these values were used to calculate the perfluorocarbon percent loading of the resuspended samples and the percentage of perfluorocarbon that sedimented over the 20-day period (Table 8). NMR spectra obtained for resuspended nanoemulsions containing 6% *w*/*v* olive oil are shown as an example to demonstrate how perfluorocarbon quantification was performed (Supplemental Figure S1). 

**Figure 5:**
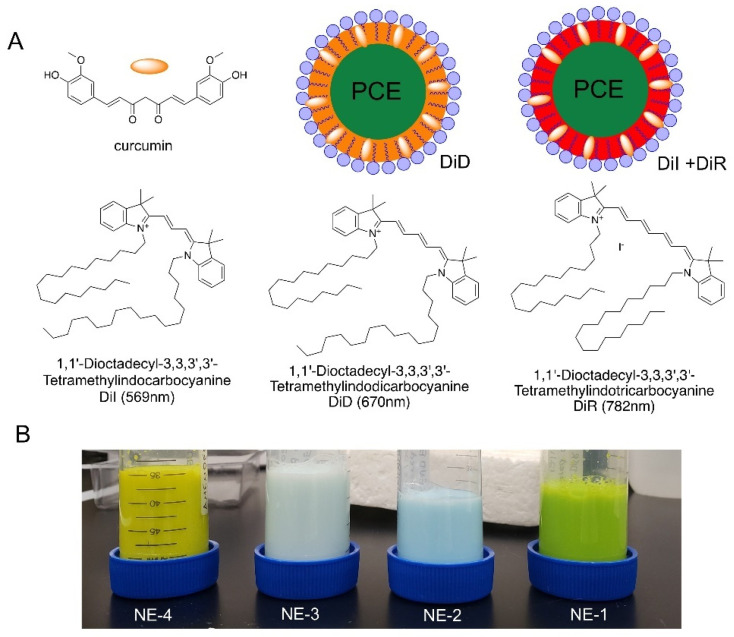
(**A**) Schematic of two-color PCE curcumin nanoemulsions. Curcumin and fluorescent dyes (DiD, DiR, and/or DiI) are incorporated into the hydrocarbon oil, which forms a corona that surrounds the PCE core. Non-ionic surfactants stabilize the hydrocarbon–water interface. (**B**) Image highlights the impact of curcumin and fluorescent dye on nanoemulsion color. From left to right: NE-4 (+CUR, +DiR); NE-3 (-CUR, +DiR); NE-2 (-CUR, +DiD); and NE-1 (+CUR, +DiD). NE-1 is a vibrant green color, while NE-4 maintains curcumin’s strong yellow color. This suggests a potential interaction between DiD and curcumin.

Each NMR sample was spiked with a known amount of trifluoroacetic acid (TFA). TFA has three chemically equivalent fluorine atoms that result in a peak at −76.0 ppm. The known amount of TFA in the sample and the integration areas of the TFA peak and a select perfluorocarbon peak were used to calculate the amount of perfluorocarbon in the nanoemulsion sample, and, subsequently, the perfluorocarbon percent loading and percent sedimentation. Additional details are provided in Section 2.7. PFOB has three chemically equivalent fluorine atoms at the end of the molecule (Figure 1) that result in a distinct peak at approximately −83.74 ppm in relation to the TFA peak (Supplemental Figure S1A). PFD is more challenging to quantify, as it consists of two diastereomers (trans and cis). CF2 groups in the trans isomer result in four doublets that range from approximately −120 to −143 ppm in relation to the TFA peak (Supplemental Figure S1B). A wide signal with two unresolved peaks is also observed at approximately −191.0 ppm. This signal is caused by the CF groups (Figure 1) and is present in both PFD isomers [16,17]. Since this signal is caused by both isomers and, therefore, accounts for 100% of PFD in the sample, this signal was used to quantify the PFD nanoemulsion samples (Figure 5B). PFPE has a strong peak at approximately −91.5 ppm (Supplemental Figure S1C) that corresponds to the CF2 groups in the monomer repeating portion of the PFPE molecule (Figure 1). The chain length is variable [11], so an estimated resonance of 32 fluorine atoms was used in all calculations, as has been previously reported [10]. PCE is the most straightforward perfluorocarbon to quantify. The symmetrical molecule has 20 chemically equivalent fluorine atoms (Figure 1) that result in a single peak at approximately −92.42 ppm in relation to TFA (Supplemental Figure S1D). NMR analysis demonstrated that nanoemulsions containing PFOB and PFD exhibit complete sedimentation of perfluorocarbon-containing droplets, as no perfluorocarbon was present in PFOB and PFD nanoemulsion samples collected prior to resuspension. PFPE and PCE nanoemulsions were found to exhibit sedimentation ranging from 21.17% (PFPE, 6% *w*/*v* olive oil, run 7) to 59.89% (PCE, 3% *w*/*v* olive oil, run 4). Across all olive oil concentrations, PFPE nanoemulsions exhibited less sedimentation than the corresponding PCE nanoemulsions. These findings are significant, as they confirm that the least stable nanoemulsions (PFOB and PFD) are the most susceptible to sedimentation, while the most stable nanoemulsions (PFPE) exhibit the lowest levels of sedimentation. Additionally, these results further strengthen our hypothesis that there is a strong correlation between nanoemulsion colloidal stability and nanoemulsion fluorescence stability. 

Nanoemulsion diameter and PDI were measured before and after resuspension to indirectly measure the extent of nanoemulsion sedimentation (Table 8). As large droplets sediment, the remaining droplets will have a smaller average diameter, and the nanoemulsion will become more homogenous, resulting in reduced PDI. PFPE nanoemulsions had the smallest values for percent diameter change and change in PDI. Accordingly, PFPE nanoemulsions also experienced the least sedimentation. PFOB and PFD nanoemulsions exhibited the largest changes in PDI, which corresponds to the complete sedimentation observed in these formulations. Interestingly, when comparing PCE nanoemulsions before and after resuspension, these formulations were found to have the largest percent diameter changes, but PDI did not increase upon resuspension. PCE nanoemulsion PDI remained comparable to the baseline value or decreased upon resuspension. The diameter and PDI changing trends were also observed when evaluating PCE nanoemulsions after exposure to accelerated stability testing or after 215 days storage at 4 °C (Figure 3 and Figure 4). Increasing diameter with minimal change in PDI is consistent with Ostwald ripening, which is the most common mechanism of nanoemulsion destabilization [18]. In Ostwald ripening, large nanoemulsion droplets increase in diameter at the expense of smaller droplets. As the number of large droplets increases, sedimentation or creaming can occur [18]. Based upon the observations in this work, we propose that PCE nanoemulsion destabilization is driven by Ostwald ripening, followed by sedimentation, while PFOB and PFD nanoemulsion destabilization is driven primarily by sedimentation alone. Nanoemulsion sedimentation results in loss of fluorescence signal, so nanoemulsions that experience the greatest levels of sedimentation will also exhibit the greatest fluorescence signal loss. 

### 3.4. Select Formulation Optimization and Curcumin Incorporation

Through the work performed thus far, we determined that the developed nanoemulsions containing PFOB and PFD were susceptible to sedimentation because these two perfluorocarbons have exceptionally high densities (>1.90 g/mL). The extensive sedimentation exhibited by these formulations resulted in unstable nanoemulsions with greater fluorescence instability. Thus, nanoemulsions containing PFPE and PCE were deemed more promising candidates for additional optimization. PCE was selected for further optimization for several reasons. First, PCE is more commercially available than PFPE. Second, PCE is preferable for ^19^F NMR and MRI applications. Third, there is a precedent for the clinical translation of PCE-containing nanomaterials. PCE poly(lactic-co-glycolic acid) nanoparticles (PCE-PLGA NPs) have been developed for applications in ^19^F MRI [19,20], ultrasound [19], and photoacoustics [21]. These PCE-PLGA NPs are currently being investigated in phase II clinical trials for labeling dendritic cells during cell therapy for stage III melanoma patients (Identifier NCT02574377) [22]. PCE nanoemulsion optimization included two stages. The first stage was used to identify a fluorescent dye that was not impacted by the presence of drug (curcumin). The second stage was used to select an appropriate oil that would maximize nanoemulsion colloidal stability, curcumin loading/stability, and fluorescent dye signal strength and stability. Nanoemulsion formulations for these two stages are summarized in Table 9. 

In stage one, two PCE nanoemulsion pairs were developed with olive oil and transcutol, one curcumin-loaded nanoemulsion (CUR-NE) and one curcumin-free nanoemulsion (DF-NE). The first pair (NE-1 and NE-2) contained the fluorescent dye DiD at a 10 µM concentration and the second nanoemulsion pair (NE-3 and NE-4) contained DiR at 10 µM. Figure 5 highlights the chemical structures of curcumin and the fluorescent dyes incorporated into the nanoemulsions and depicts the structure of the developed PCE curcumin-loaded fluorescent nanoemulsions (Figure 5A). PCE, which is both hydrophobic and lipophobic, comprises the core of the nanoemulsion droplet. The oil phase surrounding the PCE core solubilizes curcumin and/or the fluorescent dye(s). Non-ionic surfactants stabilize the nanoemulsion droplets in an aqueous continuous phase.

**Figure 6:**
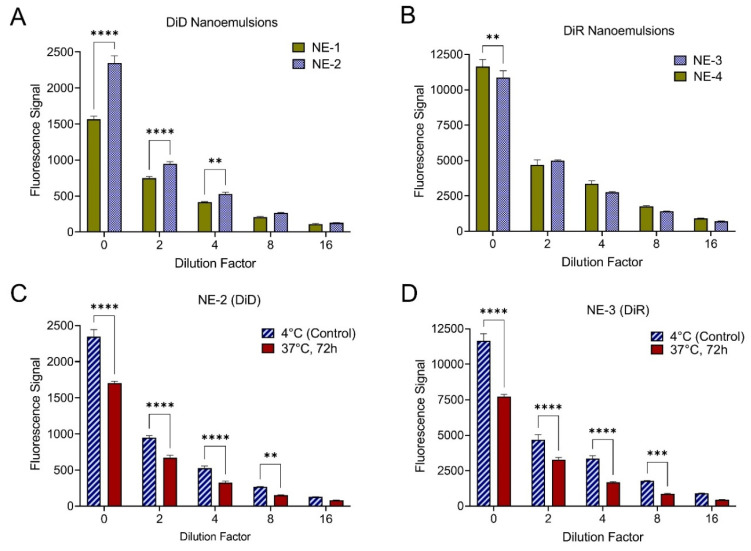
The impact of curcumin on fluorescence signal of nanoemulsions containing DiD (Panel **A**) and DiR (Panel **B**). The fluorescence signal of nanoemulsions stored at 4 °C was measured at several dilutions in de-ionized water. (**C**,**D**) The impact of 72 h incubation at 37 °C on fluorescence stability. Incubation significantly decreases the fluorescence signal of DiD (Panel **C**) and DiR (Panel **D**). ** *p*-value < 0.01; *** *p*-value < 0.001; **** *p*-value < 0.0001.

Figure 5B emphasizes the impact of fluorescent dye selection on nanoemulsion visual appearance. Curcumin-free nanoemulsions are the color of the fluorescent dye they contain, DiD (NE-2) or DiR (NE-3). The presence of curcumin adds bright yellow color to the nanoemulsion. The combination of curcumin and DiD results in a bright green color (NE-1), while the combination of curcumin and DiR is more yellow in color in comparison (NE-4). Together, this suggests that the presence of curcumin could have different impacts on each fluorescent dye. To confirm this, nanoemulsion fluorescence signal was measured at various nanoemulsion dilutions in de-ionized water to quantitatively evaluate the impact of curcumin on the fluorescence signal of DiD and DiR (Figure 6A,B). Figure 6A clearly shows that the presence of curcumin significantly reduces the fluorescence signal of DiD, while Figure 6B shows that the DiR fluorescence signal is comparable between the curcumin-loaded and curcumin-free formulations.

The stability of each fluorescent dye after nanoemulsion incubation at body temperature was also evaluated (Figure 6C,D). To accomplish this, undiluted nanoemulsion was incubated at 37 °C for 72 h. Fluorescence signal was then measured at various nanoemulsion dilutions in de-ionized water and compared to the fluorescence signal of nanoemulsion stored at 4 °C. Incubation at 37 °C for 72 h significantly reduces the fluorescence signal in both DiD (Figure 6C) and DiR (Figure 6D) nanoemulsions. Together, these results suggest that DiR is more suitable for incorporation into a curcumin-loaded PCE nanoemulsion, but that formulation adjustments should be conducted to improve the fluorescence stability of DiR. Therefore, in the second stage of nanoemulsion formulation development (Table 9), DiR nanoemulsions with different oils were developed and evaluated.

**Figure 7:**
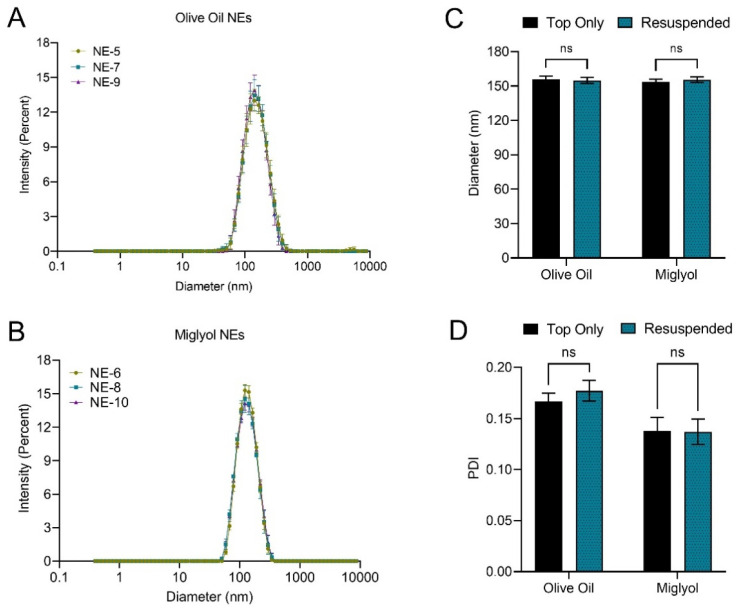
The impact of hydrocarbon oil on nanoemulsion size distribution. (**A**,**B**) Size distribution by intensity of nanoemulsions produced with (**A**) olive oil and (**B**) miglyol. (**C**,**D**) Nanoemulsion stability upon storage at 4 °C. Nanoemulsions were stored undisturbed for 20 days, after which (**C**) diameter and (**D**) PDI were measured before (top only) and after (resuspended) nanoemulsion was thoroughly mixed. ns, no significance.

In stage two of nanoemulsion formulation development, the impact of oil type on nanoemulsion colloidal stability, fluorescence stability, curcumin loading, and curcumin stability was evaluated. It was hypothesized that substitution of olive oil with miglyol may improve these properties. Therefore, a total of six curcumin-loaded nanoemulsions were developed (Table 9). Nanoemulsions were developed with no fluorescent dyes (NE-5, NE-6), with DiR at 25 µM (NE-7, NE-8), and with both DiR at 25 µM and DiI at 10 µM (NE-9, NE-10). Three nanoemulsions were developed with olive oil (NE-5, NE-7, NE-9) and three nanoemulsions were developed with miglyol (NE-6, NE-8, NE-10). Figure 7 highlights the impact of oil type on nanoemulsion colloidal stability. Nanoemulsion diameter and PDI were measured 48 h after production with dynamic light scattering. Size distribution by intensity plots is shown for nanoemulsions containing olive oil (Figure 7A) and miglyol (Figure 7B). These plots demonstrate that both olive oil and miglyol nanoemulsions can be consistently produced. The developed nanoemulsions contain 30% *w*/*v* PCE, which has a density of 1.78 g/mL. This could cause the developed nanoemulsions to be susceptible to sedimentation. To evaluate this possibility, nanoemulsions were stored at 4 °C undisturbed for 20 days, after which diameter and PDI were measured before and after nanoemulsion was thoroughly mixed/resuspended. Sedimentation of large nanoemulsions droplets would result in an observable reduction in diameter and/or PDI prior to resuspension. After the 20 days storage, no visible sediment was observed in any of the nanoemulsions. Further, both olive oil and miglyol nanoemulsions exhibited no significant change in diameter (Figure 7C) or PDI (Figure 7D) after resuspension, suggesting that nanoemulsions do not undergo significant sedimentation. This finding is highly significant, as previously developed PCE nanoemulsions experienced significant reduction in diameter upon 20 days storage (Table 8). Partial sedimentation of these formulations was visually observed, and this observation was quantitatively confirmed with ^19^F NMR (Table 8). Thus, the nanoemulsion optimization appears to have improved formulation colloidal stability.

**Figure 8:**
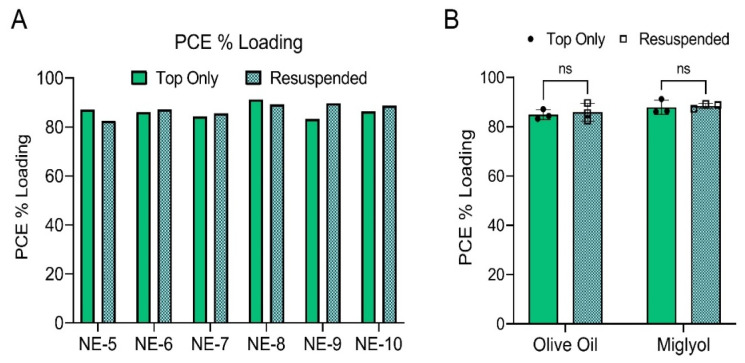
Nanoemulsion PCE loading for stage 2 nanoemulsions. Nanoemulsions were stored undisturbed for 20 days, after which PCE loading was measured before (top only) and after (resuspended) nanoemulsion was thoroughly mixed. PCE loading values are shown for (**A**) each nanoemulsion individually and (**B**) as the average of three nanoemulsions grouped by hydrocarbon oil. ns, no significance.

To confirm that nanoemulsions do not experience significant sedimentation, PCE content was quantified in nanoemulsions before and after resuspension using ^19^F NMR (Figure 8). Figure 8A shows PCE loading values for each individual nanoemulsion. Additionally, the pooled average PCE loading was calculated for the three olive oil nanoemulsions (NE-5, NE-7, NE-9) and the three miglyol nanoemulsions (NE-6, NE-8, NE-10) produced during stage 2 of formulation development (Figure 8B). A two-way ANOVA with Tukey’s multiple comparisons was performed, and it was determined that oil type had no significant impact on PCE loading, and that PCE loading did not significantly change when comparing nanoemulsions before and after resuspension. Together with the diameter and PDI data (Figure 7C,D), this strongly suggests that nanoemulsions are colloidally stable and do not sediment upon storage. Interestingly, nanoemulsions containing miglyol were found to have a significantly lower PDI compared to olive oil nanoemulsions (two-way ANOVA, *p*-value < 0.0001). Miglyol nanoemulsions had average PDI values of 0.138 and 0.137 before and after mixing, respectively, while olive oil nanoemulsions had average PDI values of 0.167 and 0.177 before and after mixing (Figure 7D). This suggests that when compared to miglyol nanoemulsions, nanoemulsions developed with olive oil could be more susceptible to Ostwald ripening and, thus, could be more susceptible to changes in diameter and/or PDI. 

**Figure 9:**
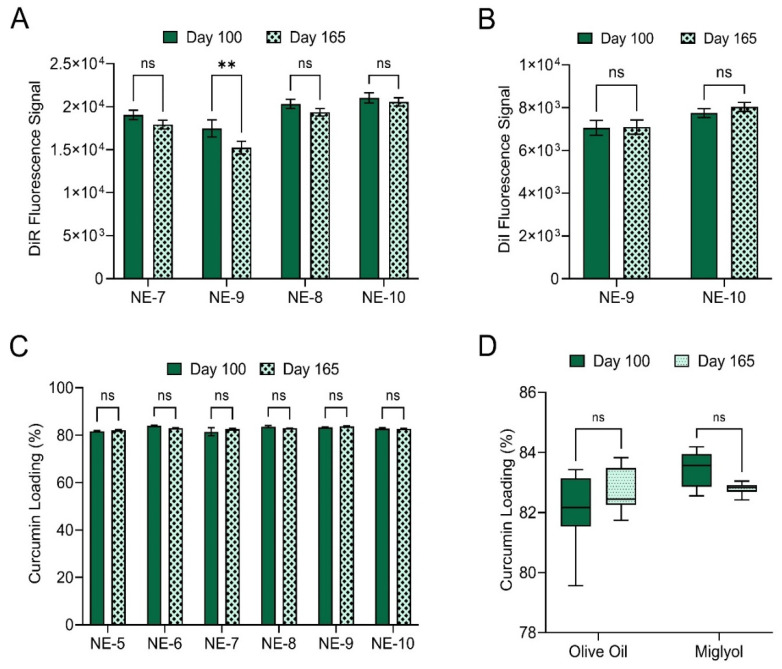
Nanoemulsion fluorescence and curcumin loading stability upon 65 days storage at 4 °C. (**A**,**B**) Fluorescence signals of stage 2 nanoemulsions containing (**A**) DiR or (**B**) DiI were measured on days 100 and 165 post manufacture. (**C**,**D**) Curcumin loading of stage 2 nanoemulsions on days 100 and 165 post manufacture. Curcumin loading values are shown for (**C**) each nanoemulsion individually and (**D**) as the average of three nanoemulsions grouped by hydrocarbon oil. ** *p*-value < 0.01; ns, no significance.

To ensure maximum performance and shelf life of the final product, it is essential that nanoemulsion fluorescence signal and curcumin loading remain unchanged upon prolonged storage. To assess this, nanoemulsion DiR signal, DiI signal and curcumin loading were evaluated on days 100 and 165 post production (Figure 9). Of the four nanoemulsions containing DiR, NE-9 was the only nanoemulsion to experience a significant decrease in DiR signal over the 65-day storage period (Figure 9A, *p*-value 0.0019, two-way t-test). Neither of the two nanoemulsions containing DiI experienced a significant change in DiI signal over the 65-day storage period (Figure 9B). Further, no significant change in curcumin loading was observed for the six curcumin-loaded nanoemulsions over the 65-day storage period (Figure 9C). Data pooling by oil type demonstrated that oil type does not have a significant impact on curcumin loading over the 65-day storage period (Figure 9D). This finding is highly significant, as previously developed PCE nanoemulsion formulations experienced significant loss of fluorescence signal upon storage at 4 °C (Supplemental Tables S1 and S2). Therefore, the nanoemulsion optimization presented in this work appears to have improved formulation fluorescence stability.

**Figure 10:**
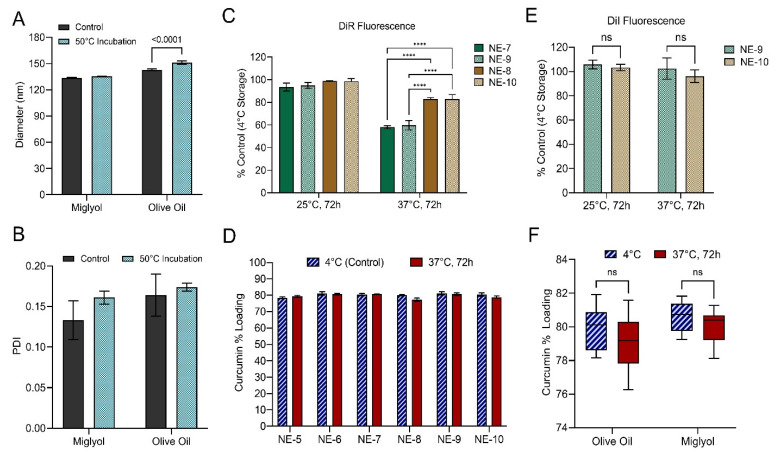
The impact of elevated temperature on nanoemulsion colloidal, fluorescence, and curcumin loading stability. (**A**,**B**) Nanoemulsion (**A**) diameter and (**B**) PDI after incubation at 50 °C for 7 days. Values are the average ± standard deviation of three nanoemulsion batches. (**C**,**E**) Nanoemulsion fluorescence signal after 72 h incubation at 25 or 37 °C. (**D**,**F**) Nanoemulsion curcumin loading after 72 h incubation at 37 °C. Curcumin loading values are shown for (**D**) each nanoemulsion individually and (**F**) as the average of three nanoemulsions grouped by hydrocarbon oil. **** *p*-value < 0.0001; ns, no significance.

To further evaluate the impact of oil type on nanoemulsion colloidal stability, fluorescence stability, and curcumin loading, nanoemulsions were subjected to elevated temperature as a form of accelerated stability testing (Figure 10). To assess the impact of oil type on nanoemulsion colloidal stability, undiluted nanoemulsions were incubated at 50 °C for 7 days (Figure 10A,B). The pooled average diameter (Figure 10A) and PDI (Figure 10B) were calculated for the olive oil nanoemulsions (NE-5, NE-7, NE-9) and miglyol nanoemulsions (NE-6, NE-8, NE-10) after 50 °C incubation and compared to the average diameter and PDI for nanoemulsions stored at 4 °C. Incubation at 50 °C for 7 days results in a significant increase in average olive oil nanoemulsion diameter (Figure 10A, two-way ANOVA, Tukey’s multiple comparisons, *p*-value < 0.0001), while average miglyol nanoemulsion diameter does not significantly change. Incubation at 50 °C does not significantly change average PDI, regardless of oil type (Figure 10B). These results complement previous findings that olive oil nanoemulsions have a significantly higher PDI compared to miglyol nanoemulsions (Figure 7D). Together, these results suggest that compared to miglyol nanoemulsions, olive oil nanoemulsions are more susceptible to diameter increase via Ostwald ripening because their higher PDI value indicates a broader range of nanoemulsion droplet sizes, which accelerates Ostwald ripening. 

To assess the impact of oil type on nanoemulsion fluorescent stability, undiluted nanoemulsions were incubated at 25 or at 37 °C for 72 h (Figure 10C,E). Fluorescent signal of nanoemulsions stored at 4 °C was used as a control in these studies. DiR fluorescent signal remained unchanged for all for DiR-loaded nanoemulsions upon 25 °C incubation, but significant DiR signal loss was observed for all nanoemulsions upon 37 °C incubation (Figure 10C). However, DiR signal loss was significantly greater in olive oil nanoemulsions (Figure 10C, all **** indicate *p*-values < 0.0001, two-way t-tests). DiI fluorescence was robust to incubation at elevated temperature. DiI signal was unaffected by incubation at 25 or 37 °C, and there was no significant difference in DiI signal when comparing olive oil NE-9 to miglyol NE-10 (Figure 10E). 

The impact of oil type on curcumin loading and curcumin stability in nanoemulsions was evaluated by exposing the undiluted nanoemulsions to body temperature (37 °C) for 72 h (Figure 10D,F). Curcumin loading was calculated after the incubation period and compared to curcumin loading of nanoemulsion stored at 4 °C. Figure 10D shows curcumin loading values for each individual nanoemulsion. Additionally, the pooled average curcumin loading was calculated for the three olive oil nanoemulsions (NE-5, NE-7, NE-9) and the three miglyol nanoemulsions (NE-6, NE-8, NE-10) produced during stage 2 of formulation development (Figure 10F). A two-way ANOVA with Tukey’s multiple comparisons was performed, and it was determined that oil type does not have a significant impact on nanoemulsion curcumin percent loading at 4 °C or at 37 °C. Further, it was determined that incubation at 37 °C for 72 h does not have a significant impact on curcumin loading. Together, these results demonstrate that nanoemulsions can be consistently produced regardless of oil type, oil type does not significantly influence nanoemulsion curcumin loading, and incubation at 37 °C for 72 h does not have a significant impact on curcumin loading. These findings are not surprising, as the solubilizer transcutol, not the oil, is the primary curcumin carrier in the developed formulations. Since oil type does not have an impact on curcumin loading and stability in the developed formulations, oil type can be selected based on other properties, such as colloidal stability and/or fluorescence stability. 

To summarize, a PCE nanoemulsion was successfully developed with optimal colloidal, fluorescence, and drug-loading stability. DiR fluorescent dye was selected due to its lack of interference with curcumin. Miglyol oil was selected because it was found to reduce nanoemulsion Ostwald ripening and fluorescence signal loss more effectively than olive oil. The optimized nanoemulsion demonstrated superior colloidal, fluorescence, and drug-loading stability, emphasizing our earlier conclusions that nanoemulsion colloidal and fluorescence stability are correlated. 

## 4. Discussion

In the presented work, a full factorial DoE was developed to study the impact of hydrocarbon (olive oil) concentration and perfluorocarbon type on nanoemulsion colloidal and fluorescent stability. Olive oil is representative natural oil with well-known antioxidant properties [23], which could complement the anti-inflammatory effects of curcumin. Miglyol was already successfully used for formulating stable drug-loaded PFC nanoemulsions [14,24]. PFOB and PFD were investigated because these perfluorocarbons have high oxygen-dissolving capabilities [25], while PCE was investigated because it has superior ^19^F NMR and MRI sensitivity [26,27]. PFPE was investigated because this compound can be chemically conjugated to fluorescent dyes, which could improve dye stability and has been shown to prevent differential tissue distribution of the dye [10]. Thus, three levels of olive oil concentration (3, 6, and 9% *w*/*v*) and four perfluorocarbons (PFOB, PFD, PFPE, and PCE) were studied, resulting in a 12-run DoE. Nanoemulsion diameter, PDI, and fluorescence signal loss were monitored after long-term storage (215 days at 4 °C) and in response to accelerated stability conditions. When evaluating nanoemulsion properties upon long-term storage, nanoemulsions could be divided into two distinct groups. Nanoemulsions containing PFOB and PFD decreased in average diameter, had larger PDI values, and exhibited the greatest fluorescence signal loss. Further, these formulations were observed to have significant levels of sedimentation when stored undisturbed. Sedimentation can be controlled by reducing the density difference between the dispersed and continuous phases, reducing droplet size, increasing viscosity, or controlling flocculation (droplets sticking to one another but not combining) [28]. Increasing nanoemulsion hydrocarbon concentration could reduce the density of the dispersed phase and, subsequently, the density difference between the dispersed and continuous phase. This was observed in this work, as PFOB and PFD nanoemulsions with the highest studied hydrocarbon content (9% *w*/*v* olive oil) exhibited the lowest PDI values and smallest changes in droplet diameter (Supplemental Tables S1 and S2). Further, MLR models demonstrated that increasing olive oil concentration reduces droplet diameter change and PDI (Figure 2, Figure 3 and Figure 4, Table 5, Table 6 and Table 7). Nanoemulsion droplet size reduction could be achieved by increasing surfactant concentration or by increasing the number of microfluidization passes during nanoemulsion manufacture. In this work, nanoemulsion processing (sonication time and the number of microfluidization passes) was minimized because in previous work, we determined that increased nanoemulsion processing resulted in decreased drug encapsulation efficiency [14]. Sonication time was found to have a more significant impact on drug encapsulation efficiency than the number of microfluidization pulses [14]. Thus, moving forward, nanoemulsions with PFOB and PFD should be produced using a higher number of microfluidization passes. Alternatively, homogenization pressure could be increased, as this would also reduce average droplet diameter. Increasing formulation viscosity could be employed in the future for local delivery applications but should not be applied to formulations that are intended for intravenous administration. Finally, flocculation could be controlled by introducing small amounts of electrolyte or nonadsorbing polymer into the nanoemulsion continuous phase [28].

Developed nanoemulsions were evaluated extensively using pre-defined critical quality attribute (CQA) and quality control (QC) testing conditions. CQA testing conditions were designed to mimic conditions nanoemulsions would be exposed to upon future in vitro and in vivo studies, thus ensuring product efficacy and safety. QC testing conditions were more extreme and were designed to enable rapid identification of unstable formulations. In the presented work, accelerated stability tests were a good indication of how nanoemulsion colloidal properties (diameter and PDI) would respond to prolonged storage at 4 °C. MLR model terms and coefficients were comparable between accelerated stability and shelf-life stability models (Figure 3 and Figure 4, Table 6 and Table 7). This finding is highly significant, as it indicates that these accelerated stability testing conditions can be used to identify suitable nanoemulsion formulations rapidly and efficiently in future projects. 

A significant challenge encountered during this work was that the vast majority of nanoemulsion formulations in the DoE failed to meet one or more fluorescence CQA specification, and none of the developed nanoemulsions met every CQA specification for diameter, PDI, and fluorescence (Supplemental Table S1). To investigate the parameters driving fluorescence instability, MLR models were developed to predict nanoemulsion percent fluorescence signal loss upon long-term storage and in response to accelerated stability conditions. The impact of perfluorocarbon type on fluorescence stability was consistent across all models, but the impact of olive oil concentration was not. In the shelf-life model, increasing olive oil concentration was determined to increase fluorescence stability (Figure 4E,F, Table 7). This is consistent with our conclusion that nanoemulsion colloidal and fluorescence stability are correlated, as increasing olive oil concentration also improves nanoemulsion colloidal stability. The conditions in the accelerated fluorescence stability tests were not harsh enough to have an impact on colloidal stability. Thus, any loss of fluorescence signal under these conditions must be driven by another mechanism. Under the accelerated stability conditions, increasing olive oil concentration was determined to decrease fluorescence stability (Figure 2C–F, Table 5). We propose that the loss of fluorescence signal in response to elevated temperature is driven by dynamic quenching. Dynamic quenching rate increases with increasing temperature [29]. This is consistent with the presented work, as nanoemulsions incubated at 25 °C for 72 h experienced comparable fluorescence loss to nanoemulsions incubated at 37 °C for 12 h. Further, molecular oxygen quenches fluorescence via dynamic quenching [30], and perfluorocarbons are known to dissolve oxygen at high concentrations [1,6]. Increasing olive oil concentration corresponded to an increase in perfluorocarbon loading (Table 8), which, subsequently, increases nanoemulsion oxygen-dissolving capability. This may explain why increasing olive oil concentration led to decreased fluorescence stability at elevated temperatures. To summarize, increasing olive oil concentration was found to improve fluorescence stability upon long-term storage, but was also found to hinder fluorescence stability at elevated temperature. Thus, hydrocarbon concentration should be carefully selected to achieve sufficient fluorescence stability upon long-term storage without compromising stability at elevated temperature. This was the rationale behind selecting a nanoemulsion with 6% *w*/*v* olive oil for further optimization and curcumin incorporation. Though both PFPE and PCE nanoemulsions demonstrated promise throughout stability testing, PCE was selected as further optimization for two reasons. First, PCE is more readily available from suppliers. Second, PCE can be detected with greater sensitivity with ^19^F NMR and MRI. Thus, the PCE nanoemulsion with 6% *w*/*v* olive oil (Table 4, run 8) was selected for further optimization and curcumin incorporation. 

In the first stage of nanoemulsion optimization (Table 9), nanoemulsions were developed with two different fluorescent dyes: DiD and DiR. The presence of curcumin was found to decrease DiD signal, as CUR-NE fluorescence signal was lower when compared to DF-NE. DiR fluorescence signal was not impacted by the presence of curcumin (Figure 6). Curcumin itself is a fluorescent molecule that is capable of quenching other fluorophores. Curcumin has a broad fluorescent range, with maximum absorbance at 420 nm, and excitation-independent emission behavior with a maximum at 525 nm [31,32]. Fluorescent nanomaterials have been developed for curcumin detection [31], including semiconductor quantum dots [33], lanthanide upconversion nanoparticles [34], fluorescent metal nanoclusters [35,36], and carbon quantum dots [32,37,38]. Through fluorescence turn-off and/or ratiometric fluorescence response, nanomaterial fluorescence loss is used to quantify curcumin, with increasing curcumin concentration resulting in increased signal loss from the fluorescent nanomaterial [31]. Thus, it is well established that curcumin can quench a variety of fluorescent materials. DiD has fluorescence excitation and emission maxima at 644 and 665 nm, respectively, while DiR excitation and emission maxima are at 750 and 780 nm, respectively. We propose that curcumin interferes with the fluorescence intensity of DiD because of the closer proximity between the DiD and curcumin fluorescence spectra. In the presented work, DiR and DiD were both acceptable options, as both dyes could be detected in vivo using NIRF. Therefore, DiR was chosen to proceed to phase 2 of formulation optimization. 

Upon discovery that both DiR and DiD dyes were unstable in olive oil-based nanoemulsions at elevated temperature (Figure 5C,D), miglyol was introduced as a potential alternative to olive oil during stage 2 of formulation development (Table 9). Miglyol was selected as a potential oil because our group has previously developed stable, fluorescent-labeled PCE nanoemulsions with miglyol [5,14,15,24]. As anticipated, miglyol nanoemulsions had improved fluorescence stability when compared to olive oil nanoemulsions (Figure 10C). Miglyol nanoemulsions also demonstrated improved colloidal stability when compared to olive oil nanoemulsions (Figure 10A). These findings are consistent with our conclusion that there is a correlation between nanoemulsion colloidal and fluorescence stability. 

Optimized PCE nanoemulsions did not experience significant sedimentation, regardless of oil type (Figure 7 and Figure 8). This complete lack of sedimentation was unexpected, albeit beneficial. PCE sedimentation of approximately 28% was observed in a comparable nanoemulsion formulation containing 30% *w*/*v* PCE (Table 8, run 8). To develop the CUR-NEs presented here, 276 µL of olive oil from this previously studied formulation were exchanged with a solution of curcumin in transcutol. Thus, it can be concluded that introduction of transcutol improved the colloidal stability of PCE nanoemulsions by reducing Ostwald ripening and subsequent sedimentation. Introduction of alcohol ethoxylates (such as transcutol) can reduce nanoemulsion Ostwald ripening by increasing the steric repulsion between nanoemulsion droplets [28]. In previous work, we also demonstrated that PCE nanoemulsions containing transcutol were resistant to Ostwald ripening when compared to nanoemulsions without PCE [14]. Thus, there appears to be a favorable stabilizing effect driven by the excipient combination of PCE and transcutol that should be capitalized on in future work. 

Though oil type (olive oil vs. miglyol) was found to have an impact on nanoemulsion colloidal stability and fluorescence stability, it did not have a significant impact on nanoemulsion curcumin loading or curcumin stability (Figure 9 and Figure 10). This is consistent with our previous work, in which we concluded that nanoemulsion celecoxib encapsulation efficiency is dependent upon solubilizer (transcutol) concentration and nanoemulsion processing parameters (e.g., sonication time) [14]. All presented formulations contained equivalent volumes of solubilizer (transcutol) and were processed using the same conditions (sonication time, and number of microfluidization passes), so it was anticipated that all formulations would have comparable curcumin loading and stability.

## 5. Conclusions

In the presented work, we developed nanoemulsions with significantly higher perfluorocarbon content (30% *w*/*v*) than previously reported formulations (14.8% *w*/*v*) to improve their potential as ^19^F MR imaging reporters. We used QbD approaches to understand the parameters that drive colloidal and fluorescence stability in these high perfluorocarbon-containing formulations. We concluded that there is a correlation between nanoemulsion colloidal stability and fluorescence stability, and we discovered that nanoemulsions experience different destabilization mechanisms based upon perfluorocarbon type. Accelerated stability studies were developed that are accurate predictors of nanoemulsion colloidal and fluorescent behavior upon long-term storage. These accelerated stability studies should be employed in future work for the rapid identification of unsuitable formulations. The developed nanoemulsions were susceptible to sedimentation and loss of fluorescence signal, but through extensive MLR modeling, we identified parameters that contribute to these complications. We used insights gained through this modeling to optimize a PCE nanoemulsion and include curcumin as a model drug into this optimal formulation. We determined that careful selection of the fluorescent dye and oil are essential to the identification of an optimal formulation with acceptable colloidal and fluorescent stability. Miglyol-based nanoemulsions outperformed olive oil-based nanoemulsions in all colloidal and fluorescent properties, while hydrocarbon oil type did not have a significant impact on curcumin loading or stability. These results corroborate our conclusion that there is a correlation between nanoemulsion colloidal stability and fluorescent stability. Regardless of hydrocarbon oil type, introduction of solubilizer (transcutol) into the nanoemulsion formulation caused the nanoemulsions to be more resistant to sedimentation than transcutol-free formulations developed in previous studies. The optimized formulation demonstrated superior colloidal, fluorescence, and drug-loading stability, and was resistant to sedimentation. In future work, the optimized high PCE-loading curcumin nanoemulsion can be evaluated as dual ^19^F MR/NIRF imaging reporter for image guided curcumin delivery. 

## Figures and Tables

**Table 1:** Summary of perfluorocarbon properties used to calculate the theoretical number of perfluorocarbon molecules that should be present in an NMR sample if the nanoemulsion was 100% perfluorocarbon loading PFCs tested are: perfluorooctyl bromide (PFOB), perfluorodecalin (PFD), perfluoro(polyethylene glycol dimethyl ether) oxide (PFPE), and perfluoro-15-crown-5-ether (PCE). * The resonant peak at −91.53 ppm corresponds to a CF2 group in the monomer repeating portion of the PFPE molecule. The chain length is variable, so the number of 32 chemically equivalent atoms is an estimate [10,11].

PFC	MW (g/mol)	ρ (g/mL)	Peak Location(s) (ppm)	# Chemically Equivalent F Atoms at Peak	Theoretical # PFC Molecules in NMR Sample
PFOB	499	1.92	−83.74	3	7.24 × 10^19^
PFD	462	1.93	−190.9, −191.3	2	7.82 × 10^19^
PFPE	1268	1.75	−91.53	~32 *	2.85 × 10^19^
PCE	580	1.78	−92.42	20	6.23 × 10^19^

**Table 2:** Summary of diameter and polydispersity index (PDI) critical quality attribute (CQA) and quality control (QC) testing performed on perfluorocarbon nanoemulsions. * Cell culture conditions: Dilute nanoemulsion 1:80 *v*/*v* in full cull culture medium. Incubate for 3 h. Following incubation, centrifuge at 1100 rpm for 5 min.

Description	CQA or QC	Diameter Specification	PDI Specification
Initial Diameter	CQA	140–180 nm	N/A
Initial PDI	CQA	N/A	<0.25
% Diameter Change or PDI Value after:			
Filtration (0.22 µm)	CQA	<±5%	<0.25
Cell Culture Conditions *	CQA	0–10%	<0.3
Storage at 4 °C for 95 days	CQA	<±5%	<0.25
Storage at 4 °C for 215 days	QC	N/A	N/A
Incubation at 80 °C for 7 days	QC	N/A	N/A

**Table 3:** Summary of fluorescence critical quality attribute (CQA) and quality control (QC) testing performed on perfluorocarbon nanoemulsions.

Description	CQA or QC	Specification
% Fluorescence Signal Loss after:		
Incubation at 25 °C for 24 h	CQA	<10%
Incubation in cell culture media at 37 °C for 2 h	CQA	<10%
Storage at 4 °C for 95 days	CQA	<10%
Incubation at 25 °C for 72 h	QC	N/A
Incubation in cell culture media at 37 °C for 12 h	QC	N/A
Storage at 4 °C for 215 days	QC	N/A

**Table 4:** Summary of study design (left columns) and resulting nanoemulsion compositions (right columns). A full factorial design of experiments was developed to study the impact of perfluorocarbon type and olive oil concentration on nanoemulsion colloidal and fluorescent stability. * Surfactant solution is 3% *w*/*v* Cremophor EL and 2% *w*/*v* pluronic P105 in water.

DoE Specifications			Nanoemulsion Compositions		
Run	PFC Type (30% *w*/*v*)	Olive Oil Concentration (% *w*/*v*)	Olive Oil Volume (mL)	PFC Volume (mL)	Surfactant Solution * Volume (mL)
1	PFOB	3	0.818	3.886	20.292
2	PFD	3	0.818	3.906	20.276
3	PFPE	3	0.818	4.286	19.896
4	PCE	3	0.818	4.213	19.969
5	PFOB	6	1.636	3.886	19.478
6	PFD	6	1.636	3.906	19.458
7	PFPE	6	1.636	4.286	19.078
8	PCE	6	1.636	4.213	19.151
9	PFOB	9	2.454	3.886	18.660
10	PFD	9	2.454	3.906	18.640
11	PFPE	9	2.454	4.286	18.260
12	PCE	9	2.454	4.213	18.333

**Table 5:** Parameter scaled coefficients, standard errors, and *p*-values for MLR models developed to predict nanoemulsion fluorescence signal (top), nanoemulsion percent fluorescence signal loss after 72 h incubation at 25 °C (center), and nanoemulsion percent fluorescence signal loss after 12 h incubation in cell culture media at 37 °C (bottom).

Baseline Fluorescence			
Term	Scaled Coefficient	Standard Error	*p*-value
Intercept	377,797	496	<0.0001
Olive Oil Concentration	10,014	607	<0.0001
PFC Type (PFOB)	−901	859	0.3290
PFC Type (PFD)	−2181		0.0386
PFC Type (PFPE)	2797		0.0139
PFC Type (PCE)	286		0.7492
72 h Incubation at 25 °C			
Term	Scaled Coefficient	Standard Error	*p*-value
Intercept	42.05	1.42	<0.0001
Olive Oil Concentration	4.92	1.74	0.0254
PFC Type (PFOB)	12.30	2.46	0.0016
PFC Type (PFD)	15.62		0.0004
PFC Type (PFPE)	−19.58		<0.0001
PFC Type (PCE)	−8.34		0.0116
12 h Incubation at 37 °C in Cell Culture Media			
Term	Scaled Coefficient	Standard Error	*p*-value
Intercept	42.47	0.63	<0.0001
Olive Oil Concentration	8.38	0.77	<0.0001
PFC Type (PFOB)	15.70	1.09	<0.0001
PFC Type (PFD)	17.58		<0.0001
PFC Type (PFPE)	−21.21		<0.0001
PFC Type (PCE)	−12.07		<0.0001

**Table 6:** Parameter scaled coefficients, standard errors, and *p*-values for MLR models developed to predict nanoemulsion PDI after exposure to cell culture conditions (top) and after storage at 4 °C for 215 days (bottom).

**PDI after Exposure to Cell Culture Conditions**			
**Term**	**Scaled Coefficient**	**Standard Error**	***p*-Value**
Intercept	0.2033	0.0028	<0.0001
Olive Oil Concentration	−0.0159	0.0034	0.0095
PFC Type (PFOB)	0.0513	0.0048	0.0004
PFC Type (PFD)	0.0500		0.0005
PFC Type (PFPE)	−0.0593		0.0002
PFC Type (PCE)	−0.0420		0.0009
Oil × PFC (PFOB)	−0.0131	0.0059	0.0896
Oil × PFC (PFD)	−0.0166		0.0476
Oil × PFC (PFPE)	0.0199		0.0279
Oil × PFC (PCE)	0.0099		0.1687
**PDI after 215 Days Storage at 4 °C**			
**Term**	**Scaled Coefficient**	**Standard Error**	***p*-Value**
Intercept	0.1792	0.0028	<0.0001
Olive Oil Concentration	−0.0111	0.0034	0.0305
PFC Type (PFOB)	0.0415	0.0048	0.0010
PFC Type (PFD)	0.0195		0.0153
PFC Type (PFPE)	−0.0518		0.0004
PFC Type (PCE)	−0.0092		0.1287
Oil × PFC (PFOB)	−0.0244	0.0059	0.0143
Oil × PFC (PFD)	−0.0109		0.1380
Oil × PFC (PFPE)	0.0086		0.2162
Oil × PFC (PCE)	0.0266		0.0106

**Table 7:** Parameter scaled coefficients, standard errors, and *p*-values for MLR models developed to predict nanoemulsion percent diameter change after 7 days incubation at 80 °C (top), nanoemulsion percent diameter change after 215 days storage at 4 °C (middle), and nanoemulsion percent fluorescence signal loss after 215 days storage at 4 °C (bottom).

**% Diameter Change after 7 Days Storage at 80 °C**			
**Term**	**Scaled Coefficient**	**Standard Error**	***p*-Value**
Intercept	0.26	1.06	0.8173
PFC Type (PFOB)	−9.37	1.83	0.0037
PFC Type (PFD)	−11.90		0.0013
PFC Type (PFPE)	2.22		0.2792
PFC Type (PCE)	19.05		0.0001
Oil × PFC (PFOB)	1.11	2.24	0.6432
Oil × PFC (PFD)	5.35		0.0630
Oil × PFC (PFPE)	3.02		0.2368
Oil × PFC (PCE)	−9.47		0.0083
**% Diameter Change after 215 Days Storage at 4 °C**			
**Term**	**Scaled Coefficient**	**Standard Error**	***p*-Value**
Intercept	−4.46	0.19	0.0002
Olive Oil Concentration	1.63	0.23	0.0057
% Signal Loss (215 days)	4.88	0.62	0.0042
PFC Type (PFOB)	−10.21	0.36	<0.0001
PFC Type (PFD)	−9.16	0.46	0.0003
PFC Type (PFPE)	1.37	0.45	0.0553
PFC Type (PCE)	17.99	0.37	<0.0001
Oil × PFC (PFOB)	0.75	0.42	0.1708
Oil × PFC (PFD)	3.47	0.42	0.0036
Oil × PFC (PFPE)	1.57	0.44	0.0384
Oil × PFC (PCE)	−5.80	0.40	0.0007
**% Fluorescence Signal Loss after 215 Days Storage at 4 °C**			
**Term**	**Scaled Coefficient**	**Standard Error**	***p*-Value**
Intercept	54.72	1.36	<0.0001
Olive Oil Concentration	−11.53	2.31	0.0155
% Diameter Change (215 days)	112.07	14.20	0.0042
PFC Type (PFOB)	73.16	8.37	0.0032
PFC Type (PFD)	66.14	6.39	0.0019
PFC Type (PFPE)	−10.63	2.55	0.0252
PFC Type (PCE)	−128.67	15.15	0.0034
Oil × PFC (PFOB)	−5.73	2.90	0.1426
Oil × PFC (PFD)	−25.09	3.64	0.0063
Oil × PFC (PFPE)	−10.65	4.06	0.0789
Oil × PFC (PCE)	41.47	5.58	0.0050

**Table 8:** Nanoemulsion sedimentation analyses. Nanoemulsion diameter, PDI, and perfluorocarbon content were assessed in nanoemulsions before and after resuspension of any sedimentation. These measurements were used to calculate nanoemulsion percent diameter change upon resuspension, change in PDI upon resuspension, perfluorocarbon loading of resuspended nanoemulsion, and percent perfluorocarbon sedimentation in nanoemulsion prior to resuspension. Perfluorocarbon sedimentation of >99% indicates that perfluorocarbon could not be detected in the NMR sample collected prior to nanoemulsion resuspension, suggesting that all perfluorocarbon had sedimented.

	Perfluorocarbon		Diameter			PDI		
Run	Loading (%)	Sedimentation (%)	Before (nm)	After (nm)	Change (%)	Before	After	Difference
1	64.78	>99	128.3	151.2	15.15	0.155	0.315	0.160
2	59.77	>99	130.3	140.8	7.46	0.167	0.246	0.079
3	92.88	38.12	157.2	163.7	3.97	0.148	0.132	−0.016
4	84.96	59.89	137.1	183.3	25.20	0.169	0.142	−0.027
5	91.69	>99	133.0	148.1	10.20	0.139	0.219	0.080
6	78.81	>99	134.4	145.2	7.44	0.126	0.227	0.101
7	82.96	21.17	160.3	163.6	2.02	0.138	0.117	−0.021
8	80.96	27.80	160.3	179.4	10.65	0.197	0.170	−0.027
9	89.28	>99	142.0	153.2	7.31	0.113	0.190	0.077
10	63.70	>99	142.2	149.3	4.76	0.125	0.173	0.048
11	92.88	32.16	172.9	172.8	−0.06	0.155	0.112	−0.043
12	90.73	56.55	156.1	183.9	15.12	0.196	0.187	−0.009

**Table 9:** Nanoemulsion formulations developed to identify the most suitable near infrared dye (stage 1) and oil (stage 2) for incorporation into a curcumin-loaded perfluorocarbon nanoemulsion. * Theoretical curcumin concentration (assumes 100% loading). ** Surfactant solution is 3% *w*/*v* Cremophor EL and 2% *w*/*v* Pluronic P105 in water.

	Olive Oil (mL)	Miglyol 812N (mL)	Transcutol (mL)	Curcumin * (mg/mL)	PCE (mL)	Surfactant Solution ** (mL)	DiD (µM)	DiR (µM)	DiI (µM)
Stage 1—Near Infrared Fluorescent Dye Selection									
NE-1	1.36	X	0.28	1.10	4.21	19.15	10	X	X
NE-2				X					
NE-3				X			X	10	
NE-4				1.10					
Stage 2—Oil Selection									
NE-5	1.36	X	0.28	1.10	4.21	19.15	X	X	X
NE-6	X	1.36							
NE-7	1.36	X						25	X
NE-8	X	1.36							
NE-9	1.36	X						25	10
NE-10	X	1.36							

## Data Availability

Not applicable.

## Supplementary Materials

The following supporting information can be downloaded at: https://www.mdpi.com/article/10.3390/pharmaceutics15041103/s1, Table S1: Summary of CQA values for each run in the design of experiments; Table S2: Summary of QC values for each run in the design of experiments; Figure S1: Example NMR spectra obtained from nanoemulsion samples. Figure S2: Curcumin HPLC calibration curves.
