# Supplementary material for: Multiple Linear Regression Predictive Modeling of Colloidal and Fluorescence Stability of Theranostic Perfluorocarbon Nanoemulsions
Source: Pharmaceutics. 2023 Mar 29;15(4):1103. doi: 10.3390/pharmaceutics15041103 (PMC10146561; doi:10.3390/pharmaceutics15041103)
Supplement: Supplementary file 1 [file pharmaceutics-15-01103-s001.zip › pharmaceutics-2230726-supplementary.pdf]

SUPPLEMENTAL INFORMATION

Multiple Linear Regression Predictive Modeling of Colloidal and Fluorescence Stability of Theranostic Perfluorocarbon Nanoemulsions

Michele Herneisey [1] and Jelena M. Janjic [1, 2, *]

[1] Duquesne University, Graduate School of Pharmaceutical Sciences, School of Pharmacy, Pittsburgh, PA 15282

* Correspondence: janjicj@duq.edu;

Supplemental Table S1: Summary of CQA values for each run in the design of experiments. Values that fail to meet the CQA specifications are italicized and underlined.

	**Baseline Values**		**% Diameter Change After:**			**PDI After:**			**% Fluorescence Signal Loss After:**		
Run	*Diameter* (140-180 nm)	*PDI* (<0.25)	*Filtration* (<±5%)	*Cell Culture Conditions* (0-10%)	*95 Days Storage at 4°C* (<±10%)	*Filtration* (<0.25)	*Cell Culture Conditions* (<0.30)	*95 Days Storage at 4°C* (<0.25)	*24h Incubation at 25°C* (<10%)	*2h Incubation at 37°C* (<10%)	*95 Days Storage at 4°C* (<10%)
1	166.1	*0.330*	-0.06	5.07	*-10.36*	*0.282*	0.284	*0.272*	*21.37*	2.67	*52.14*
2	158.8	*0.340*	-3.00	5.48	*-15.05*	0.221	0.291	0.219	*22.93*	4.93	*50.42*
3	167.8	0.155	*16.92*	*-0.12*	*-11.47*	*0.340*	0.144	0.126	3.82	-2.53	-2.21
4	149.2	0.153	2.44	1.26	9.79	0.201	0.163	0.136	8.72	1.57	*14.68*
5	164.7	0.191	0.61	6.09	*-10.55*	0.222	0.254	0.219	*23.44*	*13.98*	*45.82*
6	156.0	0.215	-0.41	6.70	-7.58	0.203	0.243	0.217	*31.19*	*13.09*	*60.82*
7	165.6	0.145	1.41	4.64	-0.44	0.138	0.136	0.145	9.54	3.65	*39.50*
8	154.8	0.144	-0.95	5.46	9.22	0.125	0.170	0.171	*18.63*	4.18	*33.63*
9	163.7	0.187	2.06	6.02	-7.44	0.177	0.226	0.187	*28.07*	*20.68*	*53.39*
10	160.6	0.195	0.69	5.41	-4.22	0.195	0.226	0.196	*26.98*	*14.78*	*50.27*
11	171.5	0.127	1.36	4.48	-1.54	0.134	0.152	0.117	9.76	4.83	*27.40*
12	167.9	0.147	-1.55	7.90	6.17	0.147	0.151	0.164	*20.56*	*10.03*	*34.56*

Supplemental Table S2: Summary of QC values for each run in the design of experiments.

	**% Diameter Change After:**		**PDI After:**		**% Fluorescence Signal Loss After:**		
Run	*7 Days Incubation at 80°C*	*215 Days Storage at 4°C*	*7 Days Incubation at 80°C*	*215 Days Storage at 4°C*	*72h Incubation at 25°C*	*12h Incubation at 37°C*	*215 Days Storage at 4°C*
1	-7.59	-14.31	0.316	0.261	48.18	47.41	72.69
2	-14.09	-15.89	0.229	0.222	50.63	51.74	76.76
3	0.93	-12.45	0.137	0.124	11.75	13.17	8.17
4	32.84	15.57	0.174	0.153	28.25	20.76	37.72
5	-10.53	-14.49	0.226	0.211	59.37	59.03	62.06
6	-13.58	-9.79	0.152	0.196	62.86	62.61	81.13
7	3.40	-1.64	0.137	0.139	28.39	23.04	66.19
8	15.02	12.30	0.210	0.173	36.98	31.82	47.37
9	-9.23	-11.34	0.194	0.190	55.48	68.08	58.62
10	-7.25	-7.39	0.189	0.178	59.51	65.81	64.08
11	3.11	-2.55	0.145	0.119	27.28	27.61	34.35
12	10.06	8.54	0.201	0.184	35.90	38.66	47.50

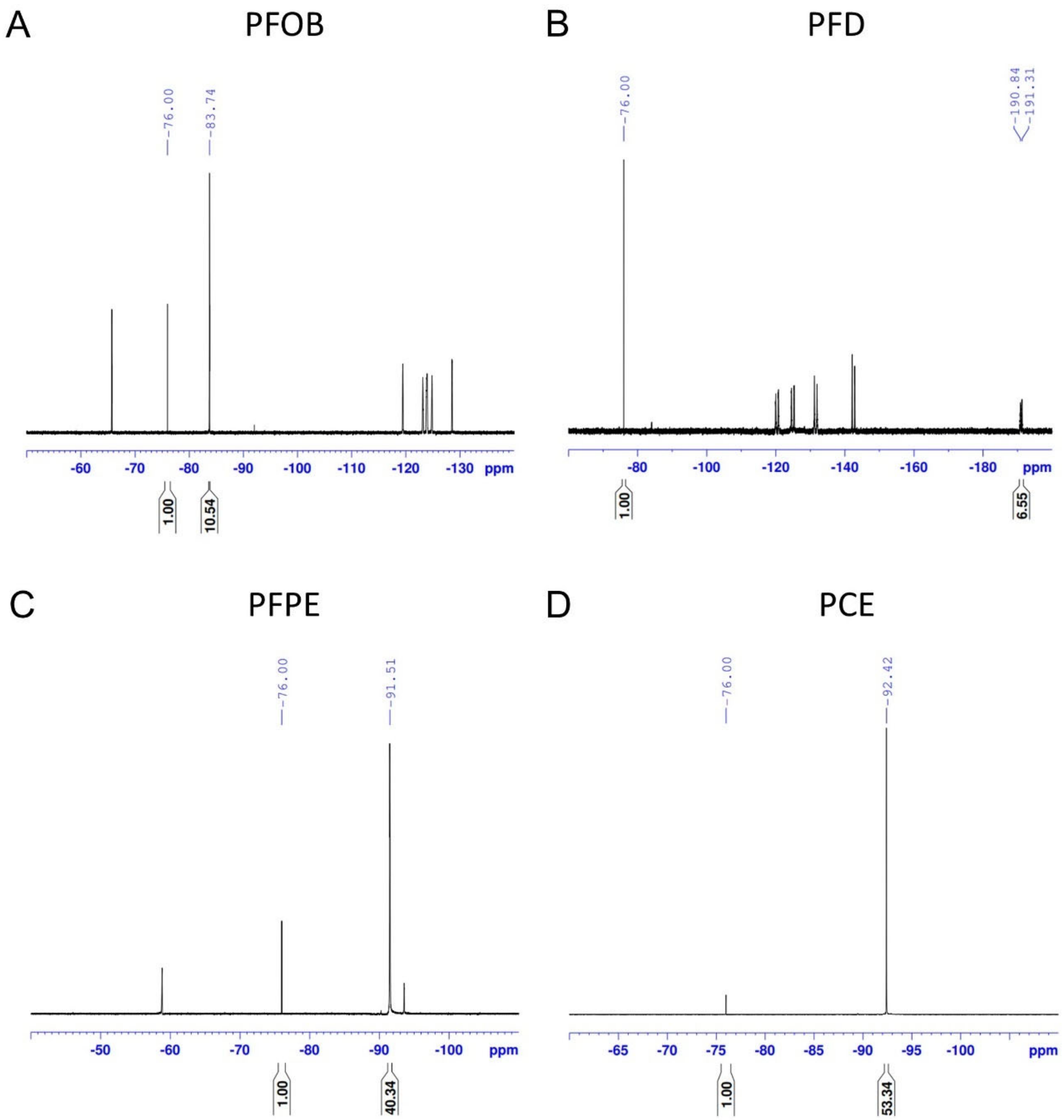

Supplemental Figure S1: Example NMR spectra obtained from nanoemulsion samples. Spectra of resuspended nanoemulsion containing 6% w/v olive oil are shown for (A) perfluorooctyl bromide (PFOB); (B) perfluorodecalin (PFD); (C) perfluoro(polyethylene glycol dimethyl ether) oxide (PFPE). (D) perfluoro-15-crown-5-ether (PCE). Peak positions (blue, top) and integration areas (bottom, black) are shown for the peaks used to quantify perfluorocarbon content.

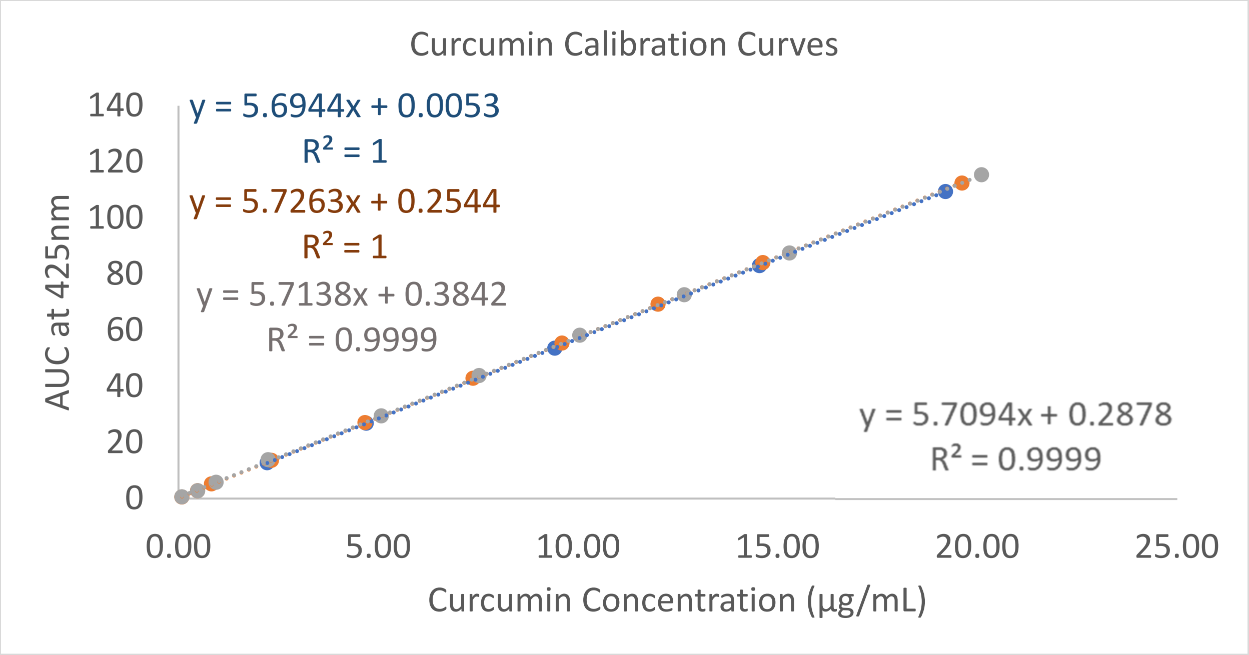

Supplemental Figure S2: Curcumin HPLC calibration curves. The calibration curve was performed in triplicate using three separately prepared stock solutions. These three curves were combined to generate a single line of best fit (bottom right equation), which was used to quantify curcumin in nanoemulsion samples.
